# Excited-state symmetry breaking is an ultrasensitive tool for probing microscopic electric fields†

**DOI:** 10.1039/d4sc04797d

**Published:** 2024-08-28

**Authors:** Bogdan Dereka, Nikhil Maroli, Yevgen M. Poronik, Daniel T. Gryko, Alexei A. Kananenka

**Affiliations:** a Department of Chemistry, University of Zurich CH-8057 Zurich Switzerland bogdan.dereka@chem.uzh.ch; b Department of Physics and Astronomy, University of Delaware Newark Delaware 19716 USA; c Institute of Organic Chemistry, Polish Academy of Sciences 01-224 Warsaw Poland

## Abstract

Microscopic electric fields are increasingly found to play a pivotal role in catalysis of enzymatic and chemical reactions. Currently, the vibrational Stark effect is the main experimental method used to measure them. Here, we demonstrate how excited-state symmetry breaking can serve as a much more sensitive tool to assess these fields. Using transient infrared spectroscopy on a quadrupolar probe equipped with nitrile groups we demonstrate both its superior sensitivity and that it does not suffer from the notorious hydrogen-bond induced upshift of the C≡N stretch frequency. In combination with conventional ground-state infrared absorption, excited-state symmetry breaking can be used to disentangle even weak specific hydrogen bond interactions from general field effects. We showcase this capability with the example of weak C–H hydrogen bonds in polar aprotic solvents. Additionally, we reveal for the first time symmetry breaking driven not by solvent but by the entropy of the pendant side chains of the chromophore. Our findings not only enhance our understanding of symmetry-breaking charge-transfer phenomena but pave the way toward using them in electric field sensing modality.

## Abstract 2

Symmetry-breaking approach exceeds the sensitivity of the vibrational Stark effect. Nitrile groups report on microscopic electric fields without interference from hydrogen-bonding interactions.

## Graphical abstract

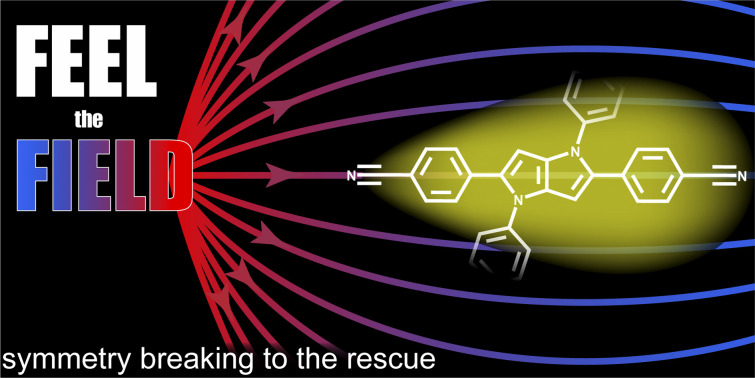

## Introduction

Excited-state symmetry breaking (ESSB) in multipolar molecules is a photophysical phenomenon in liquids that has received considerable attention over the last several years.^1–25^ First, a symmetric ground-state molecule of A–(D)_*n*_ or D–(A)_*n*_ type comprising several charge-transfer branches made of electron-donating (D) and accepting (A) moieties is promoted into an electronic excited state with a delocalized, symmetric distribution of the electron density across its branches. Then the electronic density undergoes redistribution on the ultrafast time scales resulting in, at least partially, localized asymmetric excited state. In some cases, asymmetrization can be driven until completion where the excitation localizes on a single branch.^1,4,6,7^ The extent of symmetry breaking depends on the environment and properties of the probe,^6,10,18^ such as the electronic coupling between the branches of a chromophore and the charge transfer strength that determines solvation energy. ESSB is initiated by the fluctuations of the surrounding solvent environment, and the driving force comes from the gain of the solvation energy of the final asymmetric dipolar state that compensates the loss of the excitonic interbranch coupling in the delocalized multipolar excited state.^7,10,26^ An early theoretical model of ESSB hypothesized that it is triggered by the antisymmetric vibrations and structural fluctuations of the molecule itself, along with the surrounding solvent fluctuations.^27^ However, experimentally no evidence of the role of structural fluctuations has been found to date.^3^ Several studies focused on the intramolecular structural effects of the side chains^19^ and torsional disorder^4^ but found no influence of these structural parameters on the extent of ESSB. Instead, ESSB was observed only in media where significant reaction field can be generated: in quadrupolar,^3^ dipolar,^1^ hydrogen-bonding^5^ and halogen-bonding solvents,^3^ thus providing evidence that persistent irreversible symmetry breaking is entirely driven by the solvent field. Recently, it has been observed in solvent mixtures of non-polar cyclohexane and polar acetone, where a diffusion-limited arrival of the polar component into the vicinity of the excited quadrupolar probe initiates ESSB.^8^

Elaborate theoretical models of ESSB have been developed in recent years.^26^ They treat the quadrupolar^28–36^ or octupolar^37,38^ molecules as a geometrically well-defined arrangement of excitonically coupled charge-transfer states in individual branches and a locally excited state of the core^30,36^ and take into account solute–solvent interactions,^28,30,33,35^ electron-vibration coupling,^29^ dynamic solvation^31,32^ and hydrogen bonding^34^ effects. These theoretical models successfully reproduce experimentally observed dependencies and allow for quantification of asymmetry.

Here, for the first time, we demonstrate that symmetry breaking can be driven by the pendant side chains of the quadrupolar molecule itself. We show that the outstanding sensitivity of the ESSB process to minute details in the immediate vicinity of the chromophore originates from the dependence of the asymmetry extent on the fluctuating microscopic electric fields. We further demonstrate that ESSB can serve as a useful tool to quantify these fields in various complex environments and outperforms the vibrational Stark effect (VSE) by its responsiveness. Further, our molecular probe is not susceptible to the interfering upshift of the C≡N stretch frequency induced by hydrogen bonding that commonly plagues the interpretation of nitrile VSE experiments. Our results showcase an important new application of symmetry-breaking charge transfer phenomena in strongly coupled excitonic systems where excitation does not fully collapse on a single branch. These systems can act in electric field sensing capacity with the extent of asymmetry serving as a primary metric which is directly connected to experimental observables. Given the increasing awareness of the catalytic importance of the microscopic electric fields at reaction sites,^39^ and the prospects of utilizing these fields as “smart reagents” in chemistry,^40^ our new tool to probe them will prove extremely useful in the future.

## Results

**Fig. 1:**
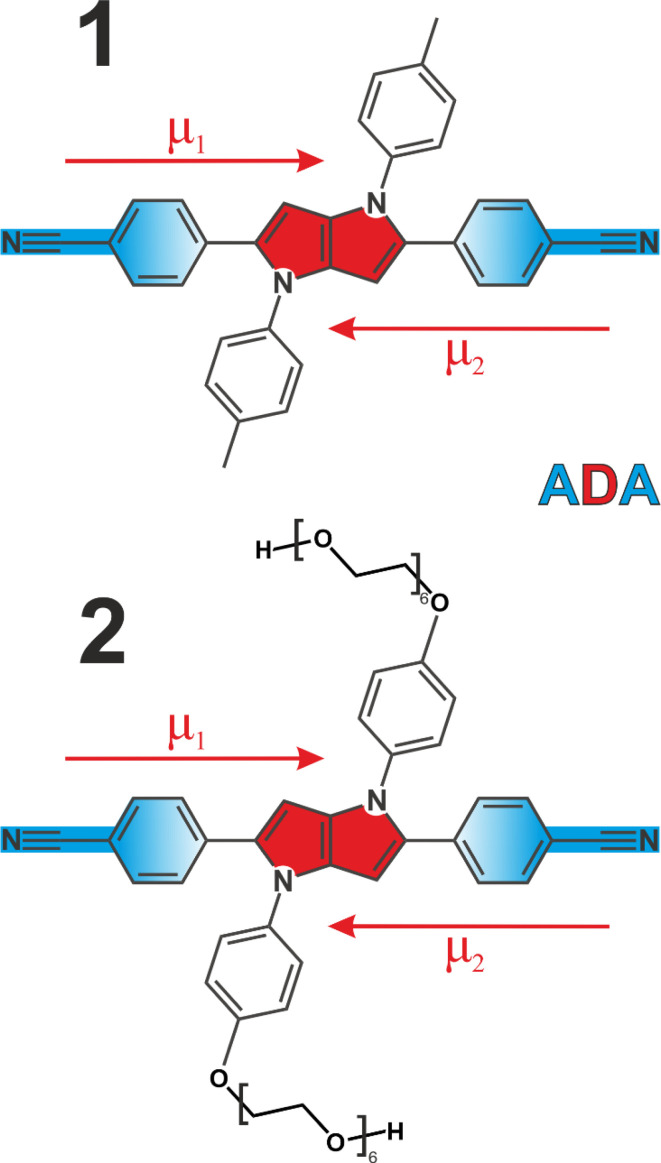
Molecular structures of quadrupolar ADA probes used in this study. The pyrrolopyrrole electron-donating core is shown in red, cyanophenyl electron acceptors are highlighted in blue. Dipole moments of individual branches (*μ*_1_, *μ*_2_) are depicted as arrows.

We employed two quadrupolar ADA type probes with a 1,4-dihydropyrrolo[3,2-*b*]pyrrole powerful electron donating core flanked by 4-cyanophenyl acceptors. This results in a strong charge transfer character along each individual branch, but zero dipole moment to the molecule overall (Fig. 1). Molecule 1 has been extensively investigated^3,5,6,41–43^ as a prototypical system for ESSB, while 2 is a modified version of 1 featuring the same chromophore but decorated with pendant hexaethylene glycol chains attached to the side phenyls at positions 1 and 4 (synthetic details are presented in ESI†). Substituents at these positions are used for modifying solubility of the chromophore without affecting its photophysical properties.^44,45^ Pegylation is a common strategy to solubilize organic molecules in aqueous environment,^46^ and we were aiming to improve the solubility of 1 in polar solvents in general and to impart water solubility in particular. We use time-resolved visible pump-IR probe (TRIR) spectroscopy as a sensitive technique to probe the extent of symmetry breaking in these molecules^1,3,5^ by monitoring the C≡N stretching frequencies in the ground and S_1_ electronic excited state.

**Fig. 2:**
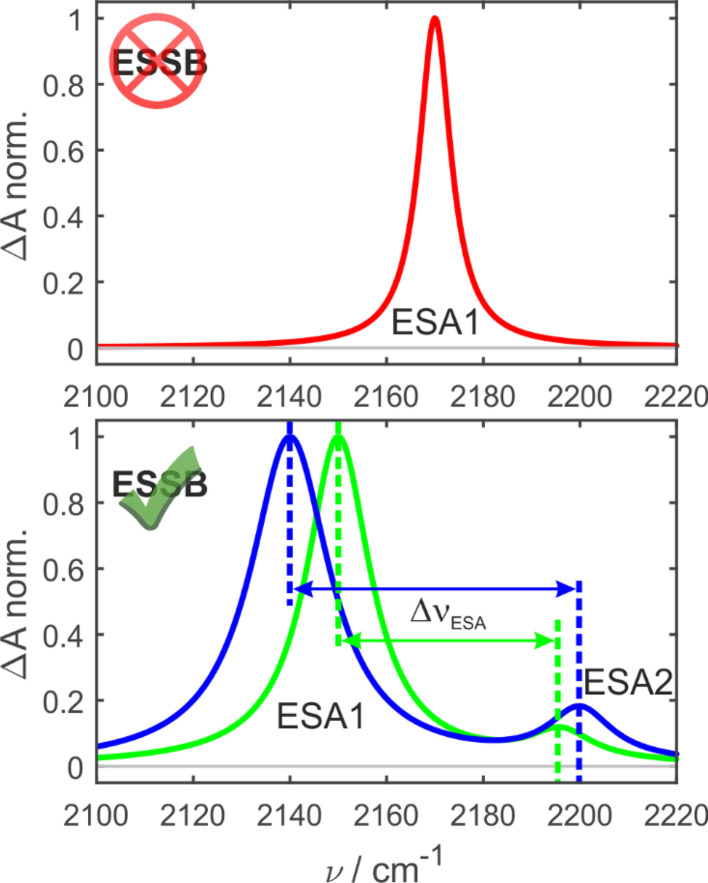
Schematic principle of the experiment. In the symmetry-preserved excited state (top) there is a single C≡N band (ESA1). Upon symmetry breaking a second band (ESA2) emerges. The splitting between the bands (Δ*ν*_ESA_) increases with asymmetry as schematically illustrated by the green and blue spectra.

In the ground-state IR spectrum, there is a single C≡N stretch band that was ascribed to the antisymmetric vibration of the two nitriles (Fig. S1†). When symmetry is preserved in non-polar media, one excited-state absorption band (ESA1) is observed in the TRIR spectrum corresponding to the same antisymmetric stretching (Fig. 2, top). Upon ESSB, ESA1 band redshifts in frequency and the second band emerges at higher frequencies (ESA2) that corresponds to the symmetric stretching vibration of the two nitriles. The splitting between these two bands (Δ*ν*_ESA_) reports on the amount of asymmetry in the excited state and thus on the progress of symmetry breaking: the larger Δ*ν*_ESA_, the more symmetry-broken is the state (Fig. 2, bottom).

**Fig. 3:**
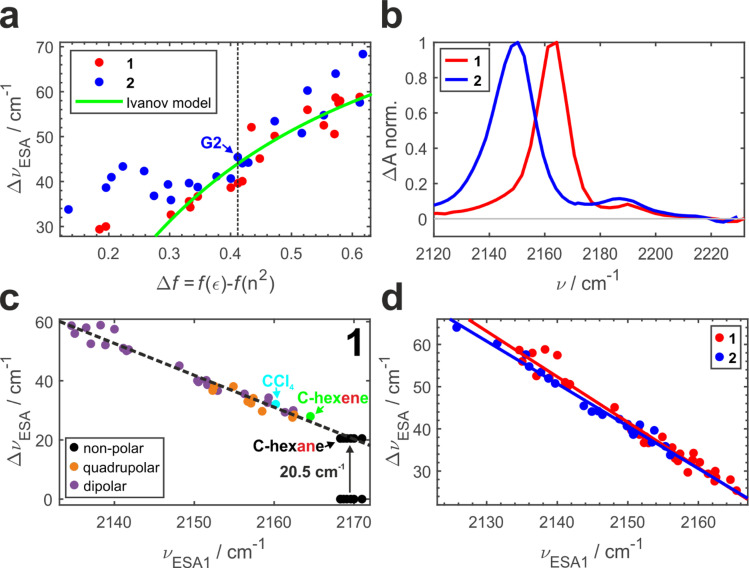
Symmetry breaking TRIR dependencies in 1 (red) and 2 (blue): (a) band splitting *vs.* the Onsager dipolar reorientation polarizability function. (b) Relaxed S_1_ state IR spectra in low polar solvents with Δ*f* = 0.195 (di-*n*-butyl ether for 1 and ethyl methyl carbonate for 2). (c) Band splitting *vs.* ESA1 peak position for 1 measured in solvents of different nature that do not participate in strong specific interactions with ADA. Solvents are colored according to their nature as strictly non-polar (black), quadrupolar (orange), dipolar (purple), as well as cyclohexene (green) and CCl_4_ (cyan). (d) Same as (c) for molecules 1 and 2 compared.

Previously, we conducted a comprehensive investigation of ESSB in 1 in 55 solvents of different nature.^3^ In 19 dipolar solvents that could not participate in specific interactions with 1, the amount of asymmetry was found to increase with the solvent reaction field that can be quantified with a simple Onsager dipolar polarizability function, Δ*f* = *f*(*ε*) − *f*(*n*^2^), where *f*(*x*) = 2(*x* − 1)/(2*x* + 1) (Fig. 3a, red markers). Each point in Fig. 3 is a result of the TRIR experiment and corresponds to the fully relaxed S_1_ state in respective solvent environment. The band splitting was accurately extracted from a lineshape analysis of the solvent-equilibrated excited state (details in Section S2.2, ESI, Fig. S7†). We note that this dependence for molecule 1 is the same as reported previously,^3^ except that we corrected the value of the Onsager function for several low polar solvents (*e.g.*, vinyl acetate and di-*n*-pentyl ether) that were erroneous due to errors in dielectric constant values reported in literature (Fig. S5†). The theoretical prediction of the Ivanov ESSB model^28^ is shown as a green line. As a new result, we show the dependence of band splitting in 2 obtained in 22 dipolar solvents. What stands out in Fig. 3a is that while the dependencies and the degree of symmetry breaking are identical in highly polar solvents (Δ*f* > ∼0.4), there are differences in medium polar (0.32 <Δ*f* < 0.4) and strong deviations in low polar (Δ*f* < ∼0.32) environments despite 1 and 2 featuring the same chromophore. In these media, dye 2 systematically displays more asymmetric electronic distribution in the excited state than 1. Fig. 3b shows the relaxed spectra of 1 and 2 obtained in solvents of identical polarity (Δ*f* = 0.195), where the amount of asymmetry is significantly larger in 2 (Fig. S6† showcases a more dipolar *tert*-butyl methyl ether with Δ*f* = 0.332). While experimental data for both molecules deviate from the Ivanov model in low dipolar solvents, the deviation is stronger for dye 2. We attribute this behavior to the influence of the pendant glycol side chains in 2. These chains are long polar tails located on the side phenyls (Fig. 1) and they can adopt many conformations, the vast majority of which will not be symmetric. This creates an asymmetric field in the vicinity of the chromophore that influences symmetry breaking together with the solvent reaction field. Originating from hexaethylene glycol, these chains are expected to provide similar field as other glymes (glycol ethers), such as diglyme (G2) or monoglyme (dimethoxyethane) solvents. Indeed, we observe that the G2 (Fig. 3a, vertical dashed line) data point is close to the position where the symmetry-breaking behavior of 1 and 2 begins to diverge. The difference increases notably upon decrease of the polarity of the surrounding environment: in less polar solvents the influence of the polar side chains is greater. One can view the effect of these pendant side chains in low polar environment as a type of preferential polar solvation forced upon a chromophore by covalent attachment of the glyme solvent molecules. As a result, it is a conflation of the solvent and side chain fluctuating fields that drives asymmetry in this case.

It is worth noting that the details of the chain conformational distribution that vary among different low polar environments determine the exact field and, in turn, the amount of asymmetry in the relaxed S_1_ state. For example, fine structure in the 0.12 < Δ*f* < 0.30 region with a pronounced peak at Δ*f* = ∼0.22 likely originates from a shift toward higher content of gauche conformations that have a higher dipolar moment and thus confer stronger field.^47,48^

While the C≡N band splitting Δ*ν*_ESA_ parameter is a direct and quantitative structural measure of symmetry breaking in the chromophore, it can be challenging to determine it accurately for weak ESSB cases due to the difficulty of fitting the low-intensity ESA2 band on top of the wing of the intense ESA1 band. Thus, in Fig. 3c we plot Δ*ν*_ESA_*versus* the position of the ESA1 band maximum, *ν*_ESA1_, and observe an almost perfectly linear relationship between these two parameters (*R*^2^ = 0.97). This is not an intuitive result because Δ*ν*_ESA_ is determined by the behavior of both ESA1 and ESA2 bands. Nonetheless, this relationship holds across all the environments free of strong specific interactions with the chromophore: not only dipolar but also quadrupolar solvents fall on the same line (orange markers, Fig. 3c). Moreover, even the solvents that belong to neither of the two classes: CCl_4_ (cyan) that is highly symmetric and formally hexadecapolar or cyclohexene (green) that does not contain any polar bonds, both obey the underlying dependence. The excited-state band position in truly non-polar media, like aliphatic hydrocarbons, deserves special attention.

In the electronic ground state, both IR absorption and Raman scattering show a single peak at the same frequency^5^ signifying that symmetric and antisymmetric C≡N stretches, that follow mutually exclusive selection rules in these two spectroscopies, occur at the same frequency. This implies that the language of symmetric/antisymmetric stretch is not correct, because each C≡N group can be treated as an independent local vibrator, and the nitrile vibrations do not couple to produce symmetric and antisymmetric combinations. In the electronic excited state in non-polar environment, only ESA1 is seen, and no symmetry breaking was detected.^3^ Based on these two observations it was concluded that in the absence of symmetry breaking in the S_1_ state, the two transitions occur at the same frequency too, and thus the band splitting is zero. However, given the linear trend in Fig. 3c, it is highly unlikely that non-polar solvents that exhibit no specific interactions with 1 and feature very low reaction fields will exhibit a drastically different Δ*ν*_ESA_*vs. ν*_ESA1_ dependence, producing data more than 20 cm^−1^ off the trendline. Instead, according to the observed linear relationship, the predicted splitting of symmetric and antisymmetric C≡N stretches in the excited state would be ∼20.5 cm^−1^. This non-zero band splitting would be very difficult to observe experimentally, and it has a purely intramolecular origin. Upon photoexcitation, the two local C≡N stretches couple and yield the antisymmetric and symmetric C≡N modes whose frequencies are no longer identical. The emergent coupling in the S_1_ state originates from electrical anharmonicity. It is negligible in the S_0_ state, where each C≡N stretch is rather weak, because each molecular branch has a small dipole moment (Fig. 1, *μ*_1,2_). However, the situation is quite different in the S_1_ state, where strong charge transfer creates a large dipole moment in each arm, even though the molecule as a whole has no dipole moment in the absence of symmetry breaking and retains its quadrupolar character. As a result, the variation of the molecular dipole moment upon (antisymmetric) vibration of two C≡N's, ∂*μ*/∂*Q*, becomes substantial, which is why the antisymmetric C≡N stretch intensity in the excited state is much stronger (compare intensities of the bleach *vs.* ESA1 in Fig. 3b). This strong modulation of the dipole moment upon vibrational displacement is more likely to be nonlinear, that is the electrical anharmonicity ∂^2^*μ*/∂*Q*^2^ is much larger, for an almost charge-separated excited state than in a weakly dipolar ground state. This is why we expect that molecules that undergo a strong increase in their dipole moment upon photoexcitation should generally exhibit this emergent coupling between the initially local vibrations. We note in passing that the original Ivanov model^28^ (Fig. 3a, green line) needs to be augmented to account for the inferred non-zero band splitting in non-polar environments.

In principle, one could measure an excited-state Raman C≡N stretch spectrum in non-polar environment and compare it to the TRIR experiment to obtain this intramolecular splitting in the absence of ESSB. In practice, however, it will be very challenging to observe due to the strong broadening of the Raman band when electronic excited-state absorption band is used for resonance enhancement as reported recently,^10^ and solubility limited if opting for the stimulated emission band resonance enhancement.

Fig. 3d combines the Δ*ν*_ESA_*vs.* ESA1 peak dependence for both 1 and 2. The large amount of experimental data (33 solvents for 1 and 22 solvents for 2) unambiguously show the identical behavior of both molecules following the same linear trend (*R*^2^ = 0.97 − 0.98, average slope ∼1.04), independent of the presence of the decorating side chains. The ESA1 band maximum can thus serve as a more accurate and robust metric of ESSB than Δ*ν*_ESA_ due to the ease of its determination. Another advantage is that it allows for comparison of the data from different solvent classes using the same scale: Fig. 3d includes data for 1 in non-dipolar solvents that cannot be placed in Fig. 3a. The extreme sensitivity of this parameter is striking. For example, the difference between cyclohexane and cyclohexene is a single π-bond, but it is enough to cause a difference of 5 cm^−1^ for *ν*_ESA1_ in these two solvents. Experimental data points for 2 never reach the low symmetry breaking regime observed for 1 (minimum Δ*ν*_ESA_ ∼32 cm^−1^ in 2*vs.* ∼24 cm^−1^ in 1). It clearly indicates that deviating behavior of 2 in medium and especially low polar solvents (Fig. 3a) is not a unique characteristic of the modified chromophore but originates from the larger surrounding field due to the entropy of the pendant side chains. Thus, these observations provide first direct spectroscopic evidence of the symmetry breaking induced not by the solvent but by the intramolecular structural factor such as pendant side chains.

Our results call for revision a previous study of Kim and coworkers^19^ that used ultrafast broadband fluorescence upconversion spectroscopy to study the effect of the side chain length on charge transfer in a symmetry-breaking quadrupolar probe using two solvents. They compared methoxyethyl *vs. n*-octyl chains that have not only different lengths but also different polarity. Our results demonstrate that swapping non-polar aliphatic tails to methoxyethyl groups is equivalent to attaching a medium polar dimethoxyethane molecule to a chromophore that exposes it to a larger effective field, which has a profound effect on the extent of symmetry breaking. Based on the time-dependent fluorescence Stokes shift, Kim and coworkers^19^ concluded that the side chains have a greater effect on the excited-state dynamics in polar dichloromethane than in less polar toluene, and that the chains control only the extent and dynamics of torsional relaxation without affecting ESSB of the quadrupolar probe. This is the opposite of what we observe with time-resolved infrared spectroscopy, which directly monitors ESSB *via* structural dynamics of the quadrupolar probe by the Δ*ν*_ESA_ metric. The occurrence and extent of symmetry breaking cannot be inferred from the fluorescence Stokes shift measurements alone, it is a too unspecific metric.^2,9,23,33,49^

**Fig. 4:**
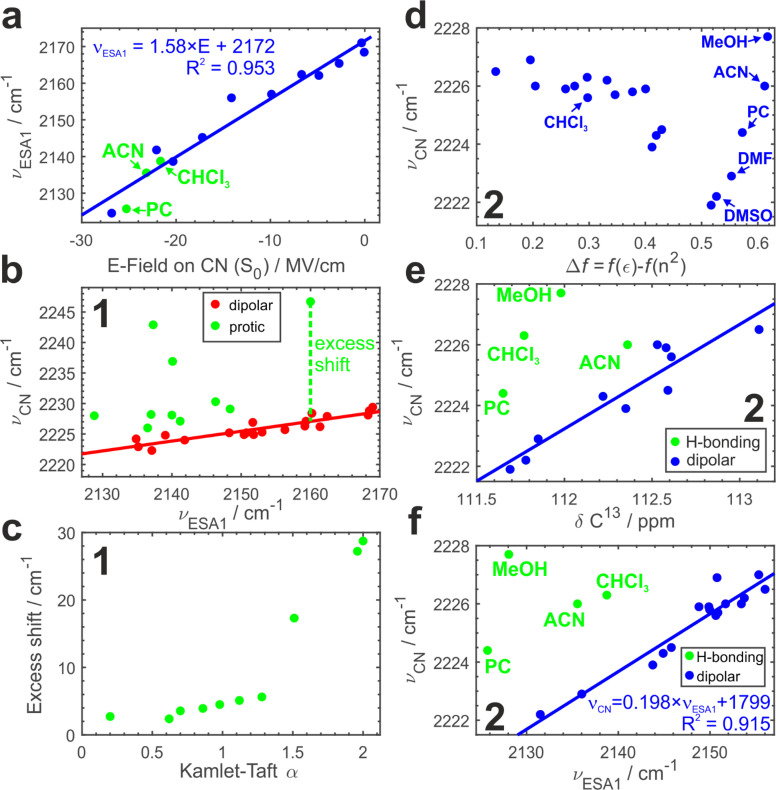
Electric field *vs.* hydrogen bonding effects: (a) dependence of the experimental ESA1 band maximum, *ν*_ESA1_, on the electric field projected onto the C≡N bond vector calculated in MD simulations. The electric field on the bond is taken as the average of the fields on the nitrile carbon and nitrogen atoms. Several hydrogen-bonding solvents evidenced by panels (e and f) are shown in green. Regression model and *R*^2^ value for the fitted line are shown. (b) Dependence of the ground-state IR absorption maximum, *ν*_CN_, *vs.* ESA1 maximum, *ν*_ESA1_, for compound 1 in aprotic (red) and protic (green) polar solvents. The red solid line is a linear fit to the aprotic data, and the green line is an illustration of the excess shift induced by H-bonding. (c) Excess shift caused by H-bonding in 1*vs.* the Kamlet–Taft *α* parameter quantifying H-bond donating ability of the solvent. (d) IR absorption maximum *vs.* the Onsager dipolar reorientation polarizability function for 2. (e) Correlation between *ν*_CN_ and ^13^C NMR chemical shift of the nitrile carbon atom in non-H-bonding (blue, linear fit is also shown) and H-bonding (green) media. (f) Same dependence as in (b) for compound 2. Regression equation and *R*^2^ value for the fitted line are shown.

To further substantiate our hypothesis that *ν*_ESA1_ acts as a proxy for the electric field, we conduct classical atomistic molecular dynamics (MD) simulations and quantify the field magnitude projected on the C≡N bond in the S_0_ state (Fig. 4a). Electric fields calculated separately on carbon and nitrogen atoms are shown in Fig. S9.† A quantitative (*R*^2^ = 0.96) linear dependence of the ESA1 band maximum on the electric field is very clear with the slope of ∼1.6 cm^−1^ (MV cm^−1^)^−1^.

### Comparison to the vibrational Stark effect

A ground-state C≡N stretch vibration gained wide recognition as an electrostatic probe^50–52^ due to its sensitivity to the surrounding field that is explained *via* the vibrational Stark effect championed by Boxer and coworkers.^53^ The VSE has become a standard method to assess microscopic electric fields at sites of local vibrational markers, *e.g.* at enzyme active sites.^50,54,55^ According to the VSE, the vibrational band maximum redshifts linearly with the field. Typically, one measures vibrational solvatochromism of the C≡N stretch in a range of solvents of increasing polarity (Fig. S1†) to calibrate the observed peak shift with the electric fields assessed from the MD simulations or a model (*e.g.*, the Onsager function).^53,55–57^ However, the VSE of nitriles is plagued by hydrogen bonding that additionally induces a blueshift of the C≡N frequency that cannot be described by the Stark effect.^58,59^ Separation of hydrogen bonding and field effects is thus required.

Using *ν*_ESA1_ as a metric for the electric field we plot in Fig. 4b the dependence of the ground-state C≡N stretch band (*ν*_CN_) of the core chromophore 1 on *ν*_ESA1_ in 29 solvents (19 of which are aprotic and 10 are protic). The data in all media separate into 2 groups. In non-hydrogen bonding ones, that engage only in non-specific interactions with the nitriles *via* electric field (Fig. 4b, red), *ν*_CN_ is linearly correlated with *ν*_ESA1_. Protic solvents additionally make hydrogen bonds with ADA that cause excess blueshift identified as a vertical offset from the underlying trendline (Fig. 4b, green). The magnitude of this excess shift is determined by the strength of the underlying hydrogen bond(s) as evidenced by a linear correlation with Kamlet–Taft *α* parameter^60^ that quantifies the H-bond donating strength of solvents (Fig. 4c). The piecewise linear form of this relationship stems from the distinctly different character of conventional protic *vs.* superprotic (polyfluorinated alcohols) solvents whose hydrogen-bond donating ability is stronger than that of water. They make strongly bound hydrogen-bonded complexes with peculiar characteristics and strongly perturb the electronic structure of ADA as described earlier.^5,43^ These results demonstrate that (i) ESSB monitored *via* the C≡N stretch mode does not suffer from the non-monotonous blueshifting effect of hydrogen bonding, (ii) combination of ESSB and ground-state IR absorption spectroscopy is capable of separating hydrogen bonding from general field effects.

Fig. 4d shows the variation of *ν*_CN_ with the Onsager function in aprotic dipolar solvents (and protic MeOH for comparison) for 2. Evidently, the relationship is complex and non-monotonous. The frequency variation reaches ∼4 cm^−1^ decreasing non-linearly from 2226 cm^−1^ in the least dipolar solvents to 2222 cm^−1^ in DMSO. After DMSO, it increases again in several highly polar solvents. Given the hydrogen-bond donating ability of methanol, the high 2228 cm^−1^ frequency in MeOH is expected, but it is not clear why strongly polar aprotic acetonitrile (ACN), propylene carbonate (PC) and DMF follow similar behavior.

Electronic S_1_ ← S_0_ absorption transition behaves analogously (Fig. S10a†). Therefore, we use the correlation of the two methods to identify a clear linear trend (Fig. S10b†) with two notable outliers (CHCl_3_ and MeOH) where the vibrational transition occurs at frequency higher than predicted by the trendline. The deviation points to hydrogen-bonding interactions between these solvents and the nitrile groups of ADA. We note that chloroform cannot be identified as an outlying point in Fig. 4d alone.

Given the complex dependence of *ν*_CN_ on Δ*f* this factor cannot solely explain its variation. Instead, we use a combination of Δ*f* and *f*(*n*^2^) with the latter describing electronic polarizability to elucidate the observed variation in *ν*_CN_. Multilinear regression on these two predictors accounts for most of the variance in the data (Fig. S10c†). Regression coefficients point to 10 times larger importance of the electronic polarizability *f*(*n*^2^) compared to that of the dipolar reorientation Δ*f*. The mean absolute value of the residuals for electronic transition amounts to 48 cm^−1^ with most of the values being in 10–30 cm^−1^ range. It highlights an often forsaken property of the Onsager model that the dipolar field works well only in media with similar electronic polarizability, that is, where the refractive index does not vary appreciably. In the current case, it is the dispersion interactions not the dipole–dipole ones that play a dominant role in ground-state vibrational solvatochromism of *ν*_CN_.

MeOH, ACN and PC in Fig. S10c† are still the most deviating solvents. Is it possible that aprotic solvents, such as acetonitrile and propylene carbonate, engage in hydrogen bonding interaction with ADA? To answer this question, we turn to another method that has been shown to be sensitive to electric fields and correct for possible hydrogen bonding effects – ^13^C NMR spectroscopy. ^13^C Chemical shift (*δ*^13^C) of the nitrile carbon atom shifts upfield to lower *δ* in more polar solvents and hydrogen bonding does not disrupt this trend,^58^ thus we could use the correlation between *δ*^13^C and ground-state IR band maximum to identify possible hydrogen bonding interactions (Fig. 4e, Section S2.4 in ESI†). Indeed, we observe a distinct linear correlation between these two parameters with most of the solvents falling onto the trendline and a few outliers above it, where *ν*_CN_ is higher as expected due to hydrogen bonding. We have already identified MeOH and CHCl_3_ as hydrogen-bonding partners (Fig. S10b†), and they are showing the strongest deviation here as well (4.5 and 4.0 cm^−1^). Additionally, we observe that PC and ACN display smaller deviations (2.4 and 1.5 cm^−1^ respectively) attributable to H-bond interaction. Therefore, we could identify very weak C–H hydrogen bonds between ADA and propylene carbonate, acetonitrile and chloroform that are present at room temperature in liquid solution and lead to only a minor shift of a few wavenumbers in *ν*_CN_. A few recent reports have shown some evidence of C–H hydrogen bond formation in chloroform^61^ and propylene carbonate.^62^

Since the core chromophore 1 displays good sensitivity and clear separation of hydrogen-bonding *vs.* electric field effects, Fig. 4f plots *ν*_CN_*vs. ν*_ESA1_ dependence for compound 2. In agreement with the results for 1, most aprotic solvents follow the identical trend as in Fig. 4b featuring the same slope (Fig. S8†). Moreover, in full agreement with the IR-NMR correlation (Fig. 4e), we can separate the hydrogen bonding effect in protic MeOH, as well as in aprotic but C–H hydrogen-bonding PC, ACN and CHCl_3_ in much the same way. This demonstrates that the NMR experiment can be substituted with TRIR to separate the impact of H-bonding.

Thus, we can rationalize complex behavior of *ν*_CN_ shown in Fig. 4d. Onsager dipolar reaction field and electronic polarizability both lead to the redshift of the peak frequency of the ground-state C≡N stretch transition with the latter being the dominant contributor. Even weak nonorthodox hydrogen bonding interactions lead to the noticeable blueshift of the C≡N stretch whose magnitude is comparable to the nonspecific effects. Importantly, all these effects are rather weak causing shifts up to a few cm^−1^ at most. According to the Onsager model, a molecule like ADA with zero dipole moment does not polarize solvent around itself and thus does not generate reaction field. Individual dipolar branches or nitrile groups themselves can weakly polarize solvent locally, which, in turn, inflicts the reaction field back onto them, but no long-range effects are expected in this case. Molecules 1 and 2 do not manifest meaningful differences in their ground-state IR spectra (Fig. S3†).

## Discussion

Our results comprehensively showcase that ESSB can serve as an excellent sensor of microscopic electric fields in the immediate vicinity of the local vibrational mode in the probe molecule. Peak frequency of ESA1 band is the most accurate and robust proxy for the extent of ESSB, as it is directly proportional to Δ*ν*_ESA_. It sensitively assesses the fields regardless of whether they originate from the solvent or decorating side chains of the molecule. Combining the slope of ESA1 field dependence (1.6 cm^−1^ (MV cm^−1^)^−1^) in Fig. 4a with that in Fig. 4f for *ν*_CN_ (0.2 cm^−1^ (cm^−1^)^−1^) we can deduce the C≡N Stark tuning rate (0.32 cm^−1^ (MV cm^−1^)^−1^). This value is in line with that reported for benzonitrile (0.36 cm^−1^ (MV cm^−1^)^−1^)^63^ and is generally on the lower side of the previously reported values for aromatic nitriles of 0.4–0.7 cm^−1^ (MV cm^−1^)^−1^.^56,64,65^ Thus, using ESA1 as a marker leads to 5 times better sensitivity compared to the conventional VSE approach. Not only the sensitivity is greater than for the VSE, but in contrast to the nitrile vibrational response in the ground state, symmetry breaking does not suffer from non-monotonous nitrile frequency shift upon hydrogen bonding.

To rationalize both sensitivity enhancement and the lack of notorious hydrogen bonding blueshift, we need to make a distinction between the mechanisms behind the field sensitivity of these two effects. The sensitivity of the VSE is determined by the change of the bond dipole moment upon vibrational excitation due to mechanical anharmonicity.^53^ This change of the dipole moment is the major factor that underlies vibrational response to the surrounding field. The electronic structure of the vibrating fragment, namely its charge distribution, remains constant. In contrast, ESSB operates in the excited electronic state and during this process the electronic structure of the excited molecule varies in response to the stronger field. The total amount of charge on both nitriles is conserved but it rebalances between the two of them during the symmetry breaking process. It is a result of this uneven distribution of the charge that ESA1 downshifts and ESA2 upshifts, because the C≡N frequency sensitively redshifts upon charge accumulation (reduction) and blueshifts (but much less) upon oxidation.^66,67^ Thus, stronger surrounding field creates more lopsided charge distribution on the nitriles. Hydrogen bonding acts similarly: its primary effect is not so much the blueshift of the C≡N frequency, but enhancement of the charge redistribution as this specific interaction helps to drive it further.^5^ As such, the blueshifting influence of the hydrogen bonding is dwarfed by the impact it produces on the electronic structure of the probe. This is a great advantage of symmetry-breaking charge transfer phenomena in such a strongly coupled system, where interbranch electronic coupling is ∼1800 cm^−1^:^42^ it prevents a complete localization but puts the system into the regime where the electronic structure of the molecule sensitively responds to the tiniest perturbations of the field. This electronic structure variation amplifies the vibrational response. This is why we can easily differentiate a very small structural change of 2 π-electrons between cyclohexane and cyclohexene *via* a 5 cm^−1^ ESA1 shift, which is comparable to the entire solvatochromism range of dicyanobenzene VSE probes.^56^ It is also the reason that we are able to assess the equilibrium fields present around the non-excited chromophore: their magnitude determines the extent of ESSB that the molecule undergoes upon photoexcitation.

In contrast, the linear dipolar Stark effect alone could not explain the vibrational solvatochromism of our probes (Fig. 4d). We demonstrated an outsized role that electronic polarizability plays in determining the response of these probes due to their low Stark rate dictated by both non-dipolar nature of the entire molecule and by the small dipole moment in each arm in the ground state. Essentially, as the molecular arms are low polar in the ground state, the dipole–dipole interactions are outweighed by the dispersion interactions that are typically much smaller. These observations highlight the importance of electronic polarizability in determining the peak shift of the C≡N mode that is typically not accounted by the VSE models.

Additionally, even weak hydrogen-bonding interaction with solvents that are usually considered aprotic and non-hydrogen-bonding (such as acetonitrile and propylene carbonate) in combination with the other two factors contributes to the complex nonlinear and non-monotonous behavior depicted in Fig. 4d. In agreement with the previous studies, combination of IR solvatochromism with other experimental methods, such as UV-vis and especially NMR spectroscopy proves useful in disentangling hydrogen-bonding from non-specific electric field effects. We note that in NMR only the chemical shift of the nitrile carbon yields a meaningful metric. Even the ipso carbon shows a more diffuse dependence, and chemical shifts of other carbons are not correlated with the field (Fig. S15†). Therefore, it is important to correctly identify the nitrile peak in ^13^C NMR spectrum if this field dependence is to be followed. Combination of vibrational solvatochromism measurements with ESSB disentangles field and hydrogen bond contributions in the same way as IR-NMR combination (Fig. 4e and f).

Boxer and coworkers have recently reported that the magnitude of the C≡N vibrational transition dipole moment can also be used to disentangle hydrogen bonding from general field effects.^68^ Thus, we quantified this observable, and it is plotted *vs. ν*_ESA1_ – our preferred field metric – in Fig. S4a.† The correlation between these two parameters is weak (*R*^2^ = 0.51), and, surprisingly, we found no relationship between *ν*_CN_ and the magnitude of the transition moment (Fig. S4b†). Thus, more systematic investigations are needed to confirm the generality of such an approach.

Additionally, we convincingly demonstrate the non-innocent nature of the pendant side chains introduced to solubilize the molecule. Entropy of these side chains drives ESSB in low polar media to a significant extent. Given the range of the extra band splitting provided by the side chains between 2 and 14 cm^−1^ depending on the solvent (Fig. 3a), the average slope of ∼1.04 for the Δ*ν*_ESA_ − *ν*_ESA1_ dependence (Fig. 3d), and ∼1.6 cm^−1^ (MV cm^−1^)^−1^ tuning rate of *ν*_ESA1_ with the field, we can estimate that they provide anywhere from 1 to 9 MV cm^−1^ field enhancement with the larger influence in low polar environments, where their effect can be dominant (*e.g.*, in organic carbonates, Δ*f* ∼ 0.2). This is the first study that identifies ESSB driven by the structural dynamics of the probe-carrying molecule itself. Our results can be compared with a recent investigation of ESSB in cyclohexane–acetone solvent mixtures, where the diffusion-limited arrival of polar component was observed to initiate ESSB process.^8^ That kind of diffusion-limited symmetry breaking was made possible by the lack of preferential solvation of the probe molecule by a polar solvent component. Instead, here, we have essentially tethered a polar chain to our quadrupolar probe and observed how it augments ESSB process.

Finally, we notice that using electric fields to describe symmetry breaking provides a compelling and physically meaningful language that unifies all sorts of interactions that were previously observed to affect ESSB process. We replace a chemical picture of particular chemical interactions, each of which requires its own description and metrics (*e.g.*, quadrupole moment for quadrupolar solvents, Kamlet–Taft *α* for H-bonding ones *etc.*) by the unified electrostatic one, where all possible non-specific and specific interactions are accounted for on the same footing and contribute toward a total symmetry breaking extent.

Electric fields are being increasingly invoked to explain a variety of catalytic processes: from the catalytic proficiency of enzymatic active sites^54,55^ to the underlying principle behind directed evolution^69^ to catalysis of (non-redox) chemical reactions^40^ or directing known chemistry to the unknown routes.^70^ As a result of intense research efforts in this direction, there is an increasing need for expanding our toolbox of instruments capable of sensitively probing microscopic fields at various molecular sites. Our work paves a new way in this direction, and we hope to stir further interest from both experimentalists and theorists alike.

## Conclusions

Our work comprehensively investigates excited-state symmetry breaking phenomenon in a prototypical quadrupolar chromophore and conceptually demonstrates its applicability for microscopic electric field sensing. Looking at the excited-state behavior of compounds 1 and 2 with ultrafast time-resolved IR spectroscopy in tens of solvents we unambiguously identified that ESSB provides an excellent sensitivity to the equilibrium fields near the molecule in the ground state that greatly exceeds that of typical Stark effect probes. The great advantage of symmetry-breaking charge transfer in a strongly excitonically coupled chromophore is that the electronic structure of the molecule sensitively responds to the tiniest perturbations in its environment. Increasing the surrounding microscopic field leads to the rebalancing of the charge between the two sides of the molecule, enhancing one at the expense of the other, which greatly amplifies the vibrational response of the C≡N markers.

We identified a structurally unequivocal and quantitative metric of symmetry breaking – the peak position of the excited-state antisymmetric C≡N stretch band, *ν*_ESA1_, that is both sensitive and robust. In conjunction with atomistic MD simulations, we quantified electric fields projected onto the C≡N bond vector and showed that they are linearly correlated with *ν*_ESA1_ metric featuring outstanding sensitivity of ∼1.6 cm^−1^ (MV cm^−1^)^−1^. Using a combination of IR, NMR, and electronic spectroscopies, we reveal that unlike frequency shifts in stationary IR spectra, *ν*_ESA1_ does not suffer from non-monotonous behavior of the C≡N stretch peak upon hydrogen bonding. Instead, combining *ν*_ESA1_ measurements with ground-state vibrational response allows for separation and quantification of a general electric field from, even very weak, hydrogen-bonding effects. We identified such weak C–H hydrogen bonds that the probe forms with aprotic polar solvents: chloroform, acetonitrile, and propylene carbonate in liquid solution at room temperature.

Additionally, we demonstrated that ESSB can be driven by the entropy of the pendant side chains attached to the probe. This is one of the unique examples where symmetry breaking is driven by an intramolecular structural factor and not by the microscopic solvent environment. Thus, we demonstrate that remote functionalization of the chromophore with solubilizing or charged groups may affect its electronic structure and can even dominate the response in some environments. This understanding will help fine-tuning the engineered internal microscopic electric fields that are considered as means of regulating chemical and catalytic activity.^63,71^

## Experimental

All experimental and computational details, as well as the additional spectroscopic data are in the ESI.†

## Author contributions

B. D. conceived the idea and performed all spectroscopic experiments. N. M. performed and analyzed MD simulations under the supervision of A. A. K. Y. P. synthesized compound 2 under the supervision of D. T. G. B. D. wrote the manuscript. All authors discussed and approved the manuscript. B. D. supervised the project.

## Conflicts of interest

There are no conflicts to declare.

## Supplementary Material

SC-015-D4SC04797D-s001

## Data Availability

The data reported in this article have been deposited to Zenodo and are available at DOI: https://doi.org/10.5281/zenodo.13372200.
