# Supplementary material for: Excited-state symmetry breaking is an ultrasensitive tool for probing microscopic electric fields
Source: Chem Sci. 2024 Aug 28;15(38):15565–76. doi: 10.1039/d4sc04797d (PMC11350400; doi:10.1039/d4sc04797d)
Supplement: SC-015-D4SC04797D-s001 [file SC-015-D4SC04797D-s001.pdf]

Supplementary Information (SI) for Chemical Science.
This journal is © The Royal Society of Chemistry 2024

SUPPLEMENTARY INFORMATION

Excited-State Symmetry Breaking Is an Ultrasensitive Tool for Probing Microscopic Electric Fields

Bogdan Dereka,[1]* Nikhil Maroli,[2] Yevgen M. Poronik,[3] Daniel T. Gryko,[3] Alexei A. Kananenka[2]

[1]*Department of Chemistry, University of Zurich, CH-8057 Zurich, Switzerland*

[2]*Department of Physics and Astronomy, University of Delaware, Newark, Delaware 19716, USA*

[3]*Institute of Organic Chemistry, Polish Academy of Sciences, 01-224 Warsaw, Poland*

* Corresponding author, e-mail: bogdan.dereka@chem.uzh.ch

Table of Contents

S1 EXPERIMENTAL DETAILS ... 2

S1.1 Synthesis ... 2

S1.2 Materials ... 4

S1.3 Methods ... 5

S1.3.1 Steady-State UV-Vis Spectroscopy ... 5
S1.3.2 Steady-State IR Spectroscopy ... 5
S1.3.3 Time-Resolved IR Spectroscopy ... 5
S1.3.4 ^{13}C Chemical Shift and 2D NMR Spectroscopy ... 6
S1.3.5 Molecular Dynamics Simulations ... 6

S2 ADDITIONAL SPECTROSCOPIC DATA AND DETAILS ... 7

S2.1 Steady-State IR Spectroscopy ... 7

S2.2 Time-Resolved IR Spectroscopy ... 9

S2.3 UV-Visible Electronic Spectroscopy ... 12

S2.4 ^{13}C NMR Spectroscopy ... 13

S3 SUPPLEMENTARY REFERENCES ... 16

S1 EXPERIMENTAL DETAILS

S1.1 Synthesis

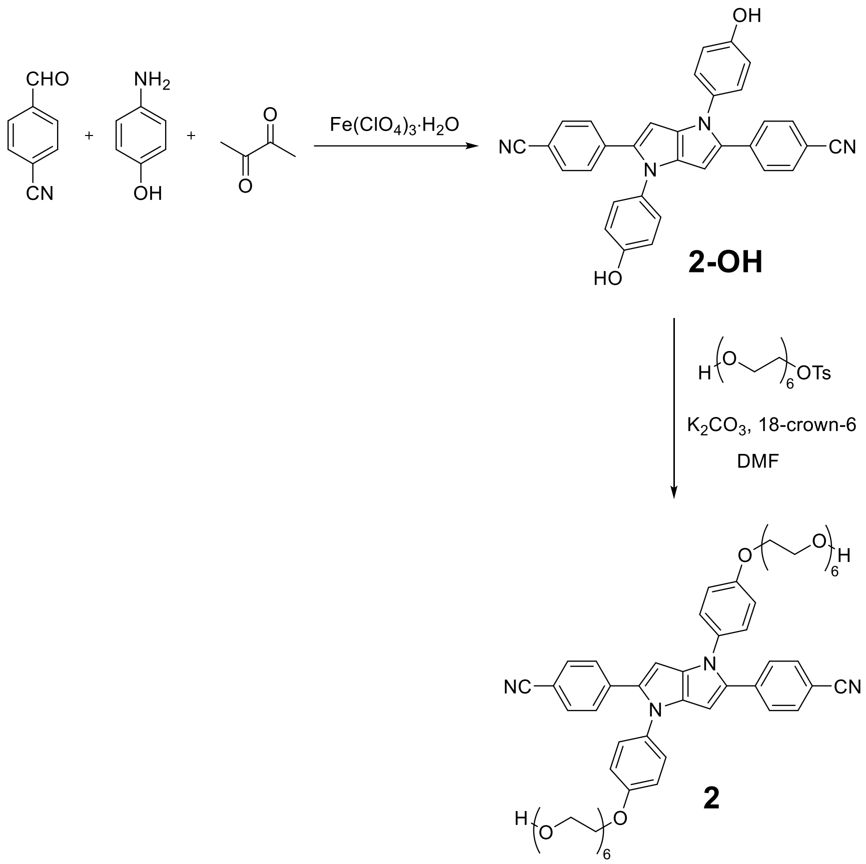

Scheme S1. Synthetic scheme to obtain compound **2**.

We synthesized pyrrolo[3,2-*b*]pyrrole modified with hexaethylene glycole moieties (compound **2**) in a two-step synthetic procedure (**Scheme S1**). A three-component process[S1] resulted in the pyrrolo[3,2-*b*]pyrrole **2-OH** with the phenol functionality which further reacted with hexaethylene glycole *p*-toluenesulfonate to yield modified pyrrolopyrrole **2**.

All chemicals were used as received unless otherwise noted. All reported ^{1}H and ^{13}C NMR spectra were collected using 500 MHz and 600 MHz spectrometers. Chemical shifts (δ ppm) were determined with TMS as the internal reference; J values are given in Hz. Chromatography was performed on silica gel (230-400 mesh). Size exclusion chromatography (SEC) was performed with Bio-Rad S-X1 Support in THF.

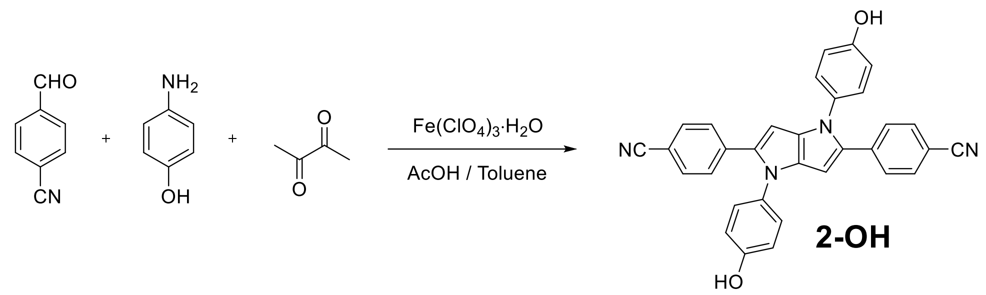

Scheme S2. Synthetic first step to access compound **2-OH**: 4,4'-(1,4-bis(4-hydroxyphenyl)-1,4-dihydropyrrolo[3,2-b]pyrrole-2,5-diyl)dibenzonitrile.

4-Aminophenol (5.45 g, 0.05 mol) and 4-formylbenzonitrile (6.55 g, 0.05 mol) were dissolved in 40 mL of the 1:1 mixture of toluene and acetic acid and the mixture was heated at 90°C for 30 min. Butan-2,3-dione (2.15 g, 0.025 mol) and iron (III) perchlorate monohydrate (0.37 g, 1 mmol) were added and the mixture was heated at 85°C with stirring for additional 20 h. On cooling the product was filtered, washed with THF and recrystallized from pyridine to give 4 g (30 %) of pure product **2-OH**. ^{1}H NMR (600 MHz, DMSO-d_6) δ 9.70 (s, 1H), 7.66 (d, J = 8.0 Hz, 2H), 7.34 (d, J = 8.2 Hz, 2H), 7.10 (d, J = 8.2 Hz, 2H), 6.84 (d, J = 8.1 Hz, 2H), 6.55 (s, 1H). ^{13}C NMR (151 MHz, DMSO-d_6) δ 156.2, 137.4, 134.7, 133.4, 132.0, 130.4, 127.4, 126.7, 119.0, 116.1, 107.8, 95.4. HRMS (APCI) calcd. for $C_{32}H_{21}N_4O_2$ 493.1665 [MH]$^+$, found 493.1666.

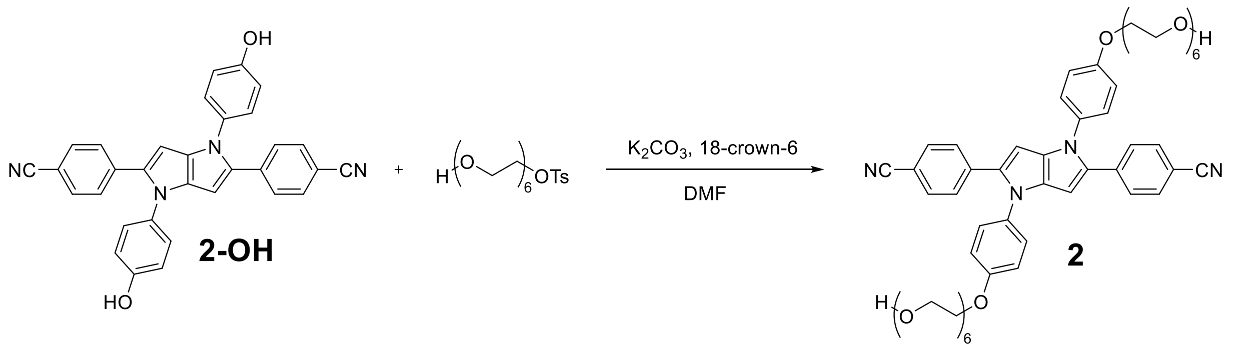

Scheme S3. Synthetic second step to access compound **2**: 4,4'-(1,4-bis(4-((17-hydroxy-3,6,9,12,15-pentaoxaheptadecyl)oxy)phenyl)-1,4-dihydropyrrolo[3,2-b]pyrrole-2,5-diyl)dibenzonitrile

Compound **2-OH** (0.67 g, 1.36 mmol), hexaethylene glycole *p*-toluenesulfonate[S2] (1.48 g, 3.4 mmol), ground potassium carbonate (0.75 g, 5.44 mmol) and 18-crown-6 (5 mg, 0.02 mmol) were heated in 20 mL of DMF at 80°C for 20 h at stirring. On cooling, the solvent was evaporated and the product was purified by the column chromatography on silica used DCM-MeOH 95:5 as the primary eluent, gradually changing to the DCM-MeOH 9:1 ratio to give 1.0 g of the crude product. The product was further eluted through SEC column in THF (Bio-Rad S-X1 Support) to give 0.55 g (40%) of pure product **2**. ^{1}H NMR (500 MHz, $CDCl_3$) δ 7.46 (d, J = 8.5 Hz, 4H), 7.26 (d, J = 8.5 Hz, 5H), 7.17 (d, J = 8.8 Hz, 4H), 6.95 (d, J = 8.9 Hz, 4H), 6.41 (s, 2H), 4.16 (t, J = 4.8 Hz, 4H), 3.89 (t, J = 4.8 Hz, 4H), 3.77 – 3.73 (m, 4H), 3.73 – 3.63 (m, 32H), 3.60 (t, 4H), 2.91 (s, 2H). ^{13}C NMR (126 MHz, $CDCl_3$) δ 157.5, 137.6, 135.2, 133.5, 132.4, 132.00, 127.8, 126.6, 119.1, 115.4, 109.00, 95.3, 72.6, 70.8, 70.6, 70.6, 70.5, 70.3, 69.7, 67.7, 61.7. HRMS (APCI) calcd. for $C_{56}H_{69}N_4O_{14}$ 1021.4810 [MH]$^+$, found 1021.4811.

S1.2 Materials

Spectroscopic data for compound **1** were used from ref. S3. Compound **2** was dissolved in solvents specified in **Table S1**. No IR spectra (either steady-state or time-resolved) could be obtained in solvents highlighted in red due to the low solubility of **2**. Upon heating **2** in water, we could reach maximum aqueous solubility of ~1.5 μM, but such a solution was not stable, and the dye fell out upon return to the room temperature. Meanwhile, for example, in di-isopropyl ether the solubility was low (4.17 μM), but the measurements could be performed reliably. Probe concentration in solution was determined from the peak absorbance of the $S_1 \leftarrow S_0$ electronic transition (around 405 nm) in the UV-visible absorption spectrum according to the Lambert-Beer law assuming the independence of the maximum molar absorption coefficient $\varepsilon_{max} = 54'000$[S4] in different solvents.[S3]

Table S1. Solvents used in the current study to prepare solutions of compound **2** (ε denotes dielectric constant, n is a refractive index).

No.	Solvent	Acronym	n	ε	$f(n^2)$	$f(\varepsilon)$	Δf
1	1,1-Diethoxymethane	DEM	1.3730	2.53	0.3711	0.5050	0.1338
2	Ethyl methyl carbonate	EMC	1.3780	2.99	0.3747	0.5702	0.1955
3	Diethyl carbonate	DEC	1.3840	3.1	0.3790	0.5833	0.2043
4	Dimethyl carbonate	DMC	1.3690	3.17	0.3682	0.5913	0.2231
5	1,1-Dimethoxyethane	DME11	1.3660	3.49	0.3660	0.6241	0.2581
6	1,1-Diethoxyethane	DEtEt11	1.3810	3.8	0.3769	0.6512	0.2743
7	Chloroform	CHCl3	1.4460	4.81	0.4211	0.7175	0.2965
8	Propyl butyrate	PrBu	1.4000	4.30	0.3902	0.6875	0.2973
9	Di-isopropyl ether	DiPrE	1.3680	4.04	0.3675	0.6696	0.3021
10	Tert-butyl methyl ether	TBME	1.3690	4.5	0.3682	0.7000	0.3318
11	Butyl acetate	BuAc	1.3940	5.1	0.3861	0.7321	0.3461
12	Ethyl propionate	EtPr	1.3840	5.65	0.3790	0.7561	0.3771
13	Propyl acetate	PrAc	1.3840	6.3	0.3790	0.7794	0.4004
14	Diglyme	G2	1.4080	7.3	0.3958	0.8077	0.4119
15	Tetrahydrofuran	THF	1.4070	7.58	0.3951	0.8144	0.4193
16	Dimethoxyethane	DME	1.3800	7.2	0.3761	0.8052	0.4291
17	Benzonitrile	BZN	1.5280	26.0	0.4709	0.9434	0.4725
18	Hexamethylphosphoramide	HMPA	1.4660	30	0.4338	0.9508	0.5170
19	Dimethyl sulfoxide	DMSO	1.4790	46.68	0.4418	0.9682	0.5264
20	Dimethylformamide	DMF	1.4310	36.71	0.4112	0.9597	0.5531
21	Propylene carbonate	PC	1.4210	64.9	0.4046	0.9771	0.5725
22	Acetonitrile	ACN	1.3440	38.8	0.3496	0.9618	0.6122
23	Methanol	MeOH	1.3280	32.6	0.3373	0.9547	0.6173
24	Water	H2O	1.3330	80.1	0.3412	0.9814	0.6402
25	Cyclohexane	CHX	1.426	2.02	0.4079	0.4048	0
26	Di-n-butyl ether	DBE	1.3970	3.1	0.3882	0.5833	0.1952

S1.3 Methods

S1.3.1 Steady-State UV-Vis Spectroscopy

Stationary UV-visible absorbance spectra were measured on Shimadzu UV-2450 spectrometer in a 1 mm quartz cuvette (Hellma) at 20°C with 1 nm spectral resolution. The background was taken against the air and neat solvent was always measured before the sample to be subtracted from the solution spectra.

S1.3.2 Steady-State IR Spectroscopy

Stationary IR spectra were measured in a transmission mode on Bruker Vertex 80v Fourier transform infrared (FTIR) spectrometer purged with nitrogen gas. The samples were held between two 2 mm thick CaF_2 windows in a homebuilt brass flow cell[5] with a laser-cut PTFE spacer of typically 250 μm thickness (50 μm for H_2O, 100 μm for DMC samples). An average of 64 individual spectra with a spectral resolution of 0.15-0.25 cm^{-1} was obtained for each measurement. The background was taken against the empty sample compartment, and solvent spectrum was taken before each solution measurement. Solvent was removed and solution injected via a PTFE tube connected to the flow cell with the help of a syringe without opening the sample compartment or touching the cell. Direct subtraction of solvent spectrum from solution spectrum was carried out without any scaling or intensity matching. This ensures acquisition of reproducible high-quality spectra even at very low absorbances of the solute (< 1 mOD).

S1.3.3 Time-Resolved IR Spectroscopy

A home-built transient UV-Vis-pump/IR-probe spectrometer was used for the TRIR measurements. Briefly, 5 kHz Ti:Sapphire regenerative amplifier (Spectra-Physics, Spitfire Pro, 800 nm, 90 fs pulse duration) pumps a homebuilt optical parametric amplifier[S6] producing mid-IR pulses centered at 2130 cm^{-1} with ~200 cm^{-1} bandwidth at full width half maximum with 100 fs duration and >2 μJ per pulse. The probe and reference beams were obtained by the front and back face reflection respectively of the IR beam off an uncoated wedged BaF_2 window. The visible pump beam was produced by frequency doubling the output of the amplifier in the BBO crystal. Pump power was attenuated with a neutral density filter and was typically set to 0.2-0.5 μJ per pulse resulting in irradiance of 0.16-0.40 mJ/cm^2. The sample was held in the same flow cell that was used for FTIR measurements. Continuous exchange of the sample during the course of the measurements was ensured by cycling the sample with a peristaltic pump (Ismatec Reglo Analog ISM795). The change in probe light absorption induced by the pump was determined using a spectrograph (Horiba Triax 190, 150 and 300 lines/mm gratings) and detected with a 2×32 pixel HgCdTe array (Infrared Associates MCT-13-64el) using home-built electronics[S7] thus determining the probe frequency axis. Between 1 to 3 grating positions were acquired depending on the sample. Adjacent spectral regions had overlapping portions to facilitate stitching of the spectral data. Spectral resolution along the probe axis was ~2.4 cm^{-1}. Multichannel referencing was used for noise suppression[S8]. The polarization dependence was determined by rotating the polarization of the pump to 0° and 90° relative to the probe using a motorized zero-order λ/2 waveplate (Thorlabs ELL14) for each pump-probe time delay. Probe polarization was fixed with the wire-grid BaF_2 polarizer mounted before the focusing parabolic mirror. The parallel ($A_{\parallel}$) and perpendicular ($A_{\perp}$) contributions to the signal were collected, and they were separately corrected by subtracting the pre-time zero pump-probe signals (t < -19 ps), and the isotropic signal was consequently determined as $A_{iso} = (A_{\parallel}+2\times A_{\perp})/3$. All spectra in this work correspond to the isotropic signals. UV-Vis and FTIR spectra of each sample were collected before and after time-resolved measurements. Integrity of the sample after time-resolved measurements was very good showing 0-5% decomposition.

S1.3.4 ^{13}C Chemical Shift and 2D NMR Spectroscopy

500 MH_Z Bruker AVANCE NEO NMR spectrometer equipped with a BBO cryoprobe was used for ^{13}C NMR measurements reported and discussed in the main text. Samples of **2** in the following (non-deuterated) solvents (see **Table S1** for acronyms) were prepared: DEM, DEC, CHCl3, PrBu, PrAc, G2, THF, DME, HMPA, DMSO, DMF, PC, ACN, MeOH at a few mM concentration (or less for the least polar solvents, such as DEM, DEC). A glass capillary filled with a D_2O solution of 3-(trimethylsilyl)propionic-2,2,3,3-d_4 acid sodium salt (TSP-d_4-Na) was inserted into a sample NMR tube as a reference and deuterium lock for all ^{13}C chemical shift and 2D NMR measurements.

S1.3.5 Molecular Dynamics Simulations

Amber force field was used for all molecules. The parameterization of ADA and solvents was performed using the restrained electrostatic potential (RESP) method. The structures of solvent molecules were obtained from PubChem Database, and the structure of ADA was built and optimized. Ground-state geometries of all solvents were optimized using density function theory (DFT) with the CAM-B3LYP functional[S9] and 6-31G(d,p) basis set using the Gaussian09 package.[S10] Electrostatic potential map for the molecules obtained using the same level of theory, RESP charges, and force field parameters were derived using a generalized Amber force field (GAFF) with AmberTools23.

All-atom molecular dynamics simulations of ADA with ground- and excited-state charges in various solvents were performed for 100 ns using generalized Amber force field. All simulations were performed in the NPT ensemble with a time step of 2 fs without any restraints. Prior to the production run, the system was minimized using the steepest descent algorithm and equilibrated in the NVT ensemble for 20 ns. The trajectories were analyzed using the final 50 ns of the production run. The pressure was controlled using a stochastic cell rescaling algorithm[S11] at 1 atm and the temperature was maintained at 300 K using a V-rescale thermostat.[S12] Electrostatic interactions were calculated using the Particle mesh Ewald method[S13] with a non-bonded cut-off distance of 0.9 nm. All bonds involving hydrogen atoms were constrained using the LINCS algorithm[S14] and periodic boundary conditions were applied in all the three dimensions. The coordinates were saved every 10 ps. Simulations were performed using GROMACS 2023.2.[15] Analysis was performed using in-house scripts.

The electric field on the N atom of the nitrile group projected on the direction of the C≡N bond was calculated using the following formula

$$E_N = \hat{r}_{CN} \cdot \sum_{j=1}^{N} \frac{q_j}{r_{jN}^2} \hat{r}_{CN}$$

where $\hat{r}_{CN}$ is the unit vector along the C≡N bond, q_j and r_j are the charge of jth atom and the distance between the jth atom and the nitrogen atoms of the nitrile group; and the sum runs over all solvent atoms. An analogous equation was used to calculate the electric field on the carbon atom.

S2 ADDITIONAL SPECTROSCOPIC DATA AND DETAILS

S2.1 Steady-State IR Spectroscopy

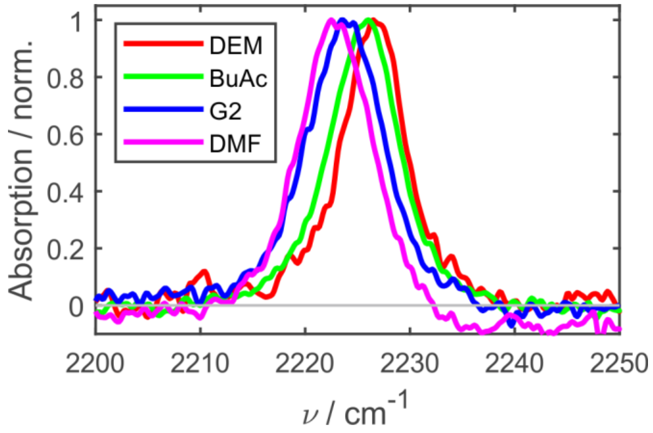

Figure S1. Steady-state FTIR spectra of **2** in a few exemplary solvents (see **Table S1**). A single band corresponding to the local C≡N stretch mode is seen. A small but noticeable solvatochromism of the vibrational transition is observed (see **Fig. 4a** in the main text).

To extract accurate band positions from the FTIR spectra, the experimental spectra were converted to the molar absorption coefficient spectra, which were fit to the Gaussian or Lorentzian lineshape in the region of interest (C≡N stretch). The background was usually fit to the linearly sloping function or an offset. Its effect was negligible. Example of such a fit is shown in **Fig. S2** for diglyme (G2). Fit quality is excellent, and both Gaussian and Lorentzian fits provide typically identical peak position estimates within 0.1 cm^{-1}.

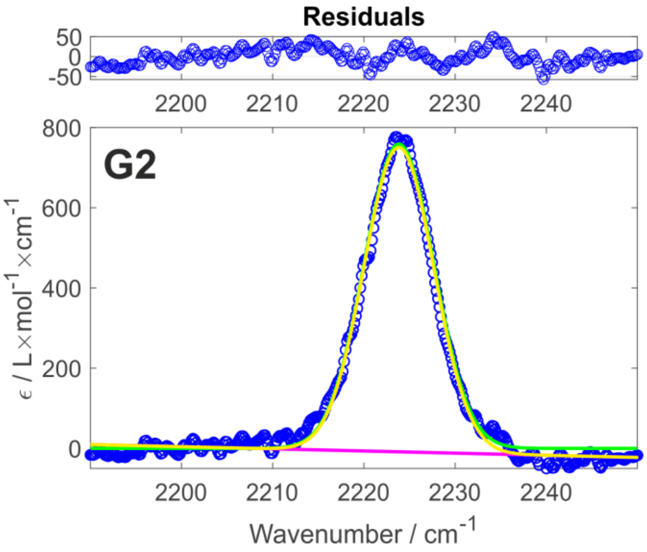

Figure S2. Experimental molar absorption coefficient spectrum of **2** in diglyme (G2) (blue markers) along with the fit (yellow line) composed of a Gaussian lineshape (green line) superimposed on a weak linearly sloping background (magenta), residuals are shown at the top.

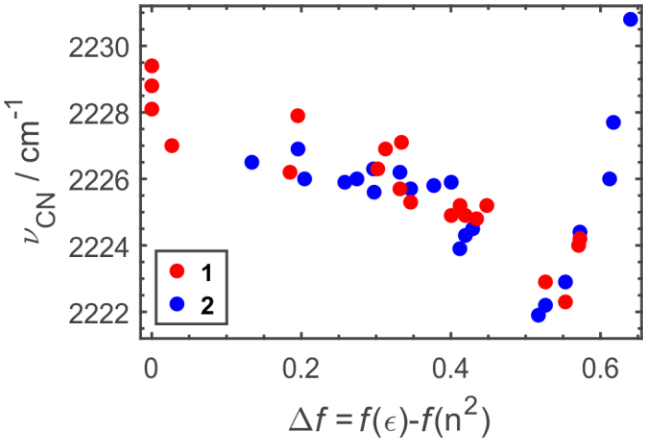

Figure S3. Maximum of the ground-state IR absorption of **1** (red) and **2** (blue) vs. Onsager dipolar reorientation polarizability function. While FTIR spectra were used for **2**, fitted minimum of the ground-state bleach signal was used for **1**, as its FTIR spectra were not measured in all the reported solvents. This contributes to slightly higher noise on the data for **1** due to the weakness of the bleach feature in TRIR spectra.

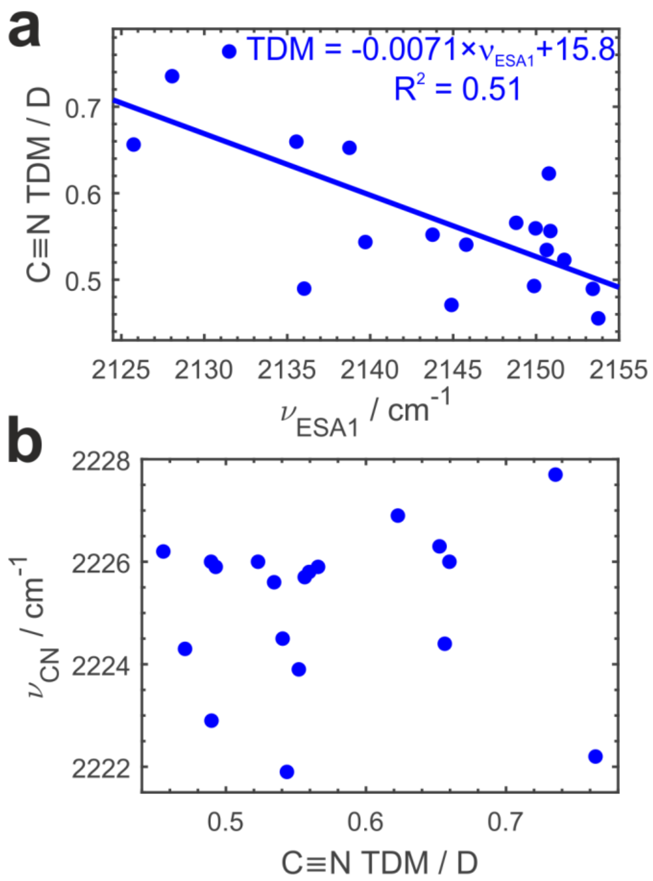

Figure S4. Absolute value of the vibrational transition dipole moment of the CN stretch transition plotted vs. ESA1 peak position – our preferred field metric (**a**) and ground-state IR band maximum (**b**). Regression equation and R^2 coefficient are shown for panel **a**.

S2.2 Time-Resolved IR Spectroscopy

Lineshape analysis of TRIR spectra was carried out by fitting a time-resolved spectrum at each time delay to a sum of a Lorentzian/asymmetric Lorentzian[S16] lineshape of ESA1, a Gaussian for ESA2 and ground-state IR absorption profile for the bleach. Various lineshape functions were attempted for ESA1 including a Lorentzian, asymmetric Lorentzian, Voigt, Gaussian, or asymmetric Gaussian. The best results are obtained when using Lorentzian/asymmetric Lorentzian options, and the differences between them are minor (**Fig. S7**). As a result, consistent usage of the same lineshape function across all of the solvents and for both probe molecules ensures the accurate extraction of the peak position parameters.

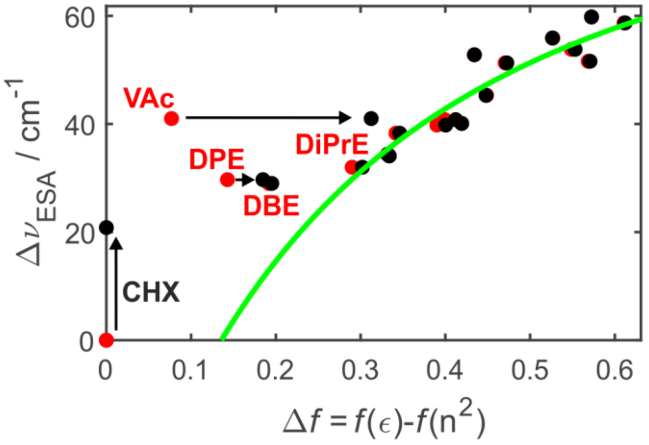

Figure S5. Band splitting as a function of the Onsager polarity function for **1** in dipolar solvents. This figure is a correction of **Figure 3** in ref. S3. The old version is shown with red markers, and the corrected values are shown with black ones. The most dramatic change in the value of the Onsager function occurs for vinyl acetate (VAc), which was erroneously considered as a low polar solvent (the wrong value of dielectric constant was 2.3), whereas in reality it is a medium polar solvent with dielectric constant of 4.5. Analogously, di-n-pentyl ether (DPE) has been corrected to have dielectric constant of 3.1 instead of 2.77. Overall, this correction removes the outlying values and puts all of the observed values on the trendline (black markers). Cyclohexane (CHX) is a special case, and correction of the value of its band splitting is discussed in the main text.

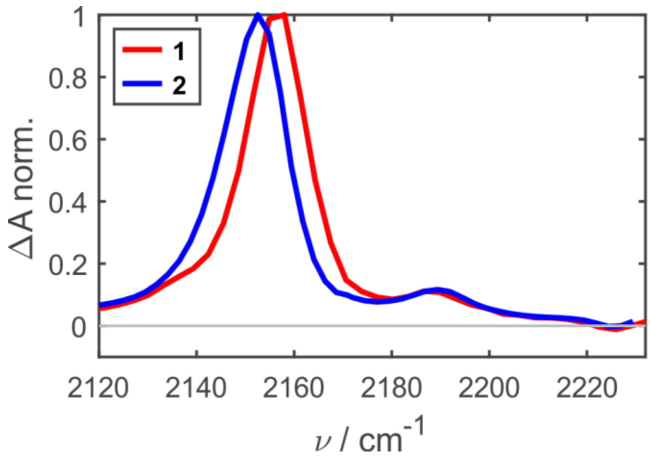

Figure S6. TRIR relaxed S_1 spectra of **1** and **2** in the medium polar tert-butyl methyl ether (TBME) ($\Delta f = 0.332$).

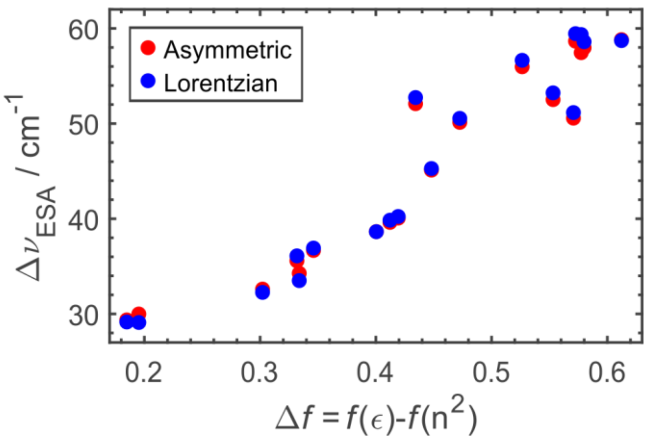

Figure S7. Comparison of the two different lineshapes taken to fit TRIR spectra: Lorentzian (blue markers) that was used previously in ref. S3 or asymmetric Lorentzian[S16] (red markers) used in the current work. The difference in the resulting curve depicting the dependence of symmetry breaking on the Onsager function is very small. Hence, change in the fitting lineshape adopted in this work does not affect the conclusions.

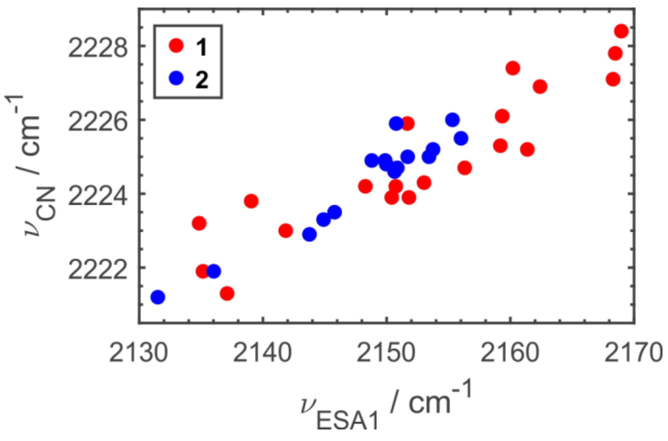

Figure S8. Maximum of the ground-state IR absorption of **1** (red) and **2** (blue) vs. ESA1 peak position – our preferred field metric. While FTIR spectra were used for **2**, fitted minimum of the ground-state bleach signal was used for **1**, as its FTIR spectra were not measured in all the reported solvents. This contributes to the higher noise on the data for **1** due to the weakness of the bleach feature in TRIR spectra. Nevertheless, it is clear that the dependence is identical for both molecules.

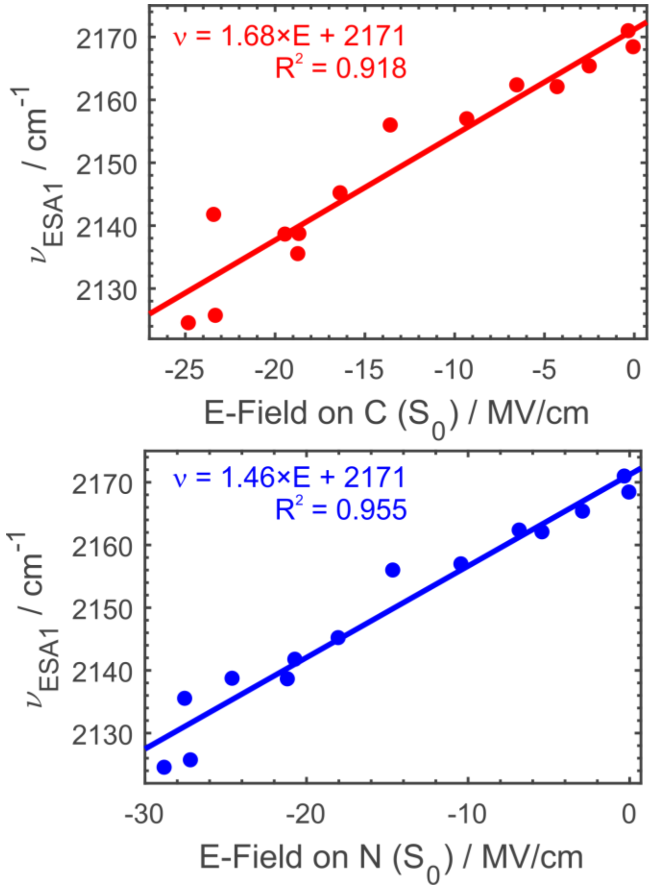

Figure S9. Dependence of ESA1 band position on microscopic electric field calculated at the nitrile's carbon (top) and nitrogen (bottom) atoms with GAFF as described in S1.3.5. Linear fits and correlation coefficients are shown.

S2.3 UV-Visible Electronic Spectroscopy

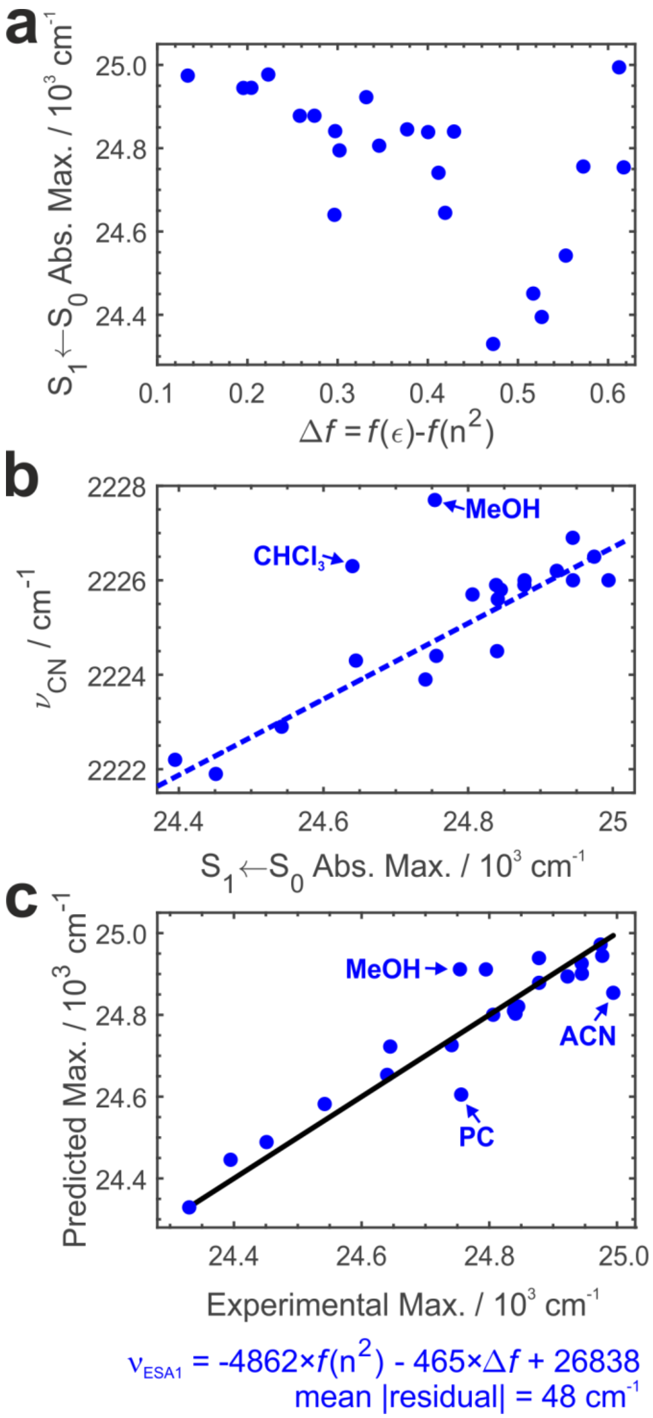

Figure S10. Electronic ground-state metrics of 2: 1. Lowest electronic absorption transition vs. the Onsager dipolar function. **2.** Correlation between the frequencies of the electronic and vibrational transitions shown in **1** and **Fig. 4d**. **3.** Results of the multilinear regression on the electronic transition absorption maximum using Onsager function Δf and electronic polarizability $f(n^2)$ as predictors. Several most deviating solvents are labelled.

S2.4 ^{13}C NMR Spectroscopy

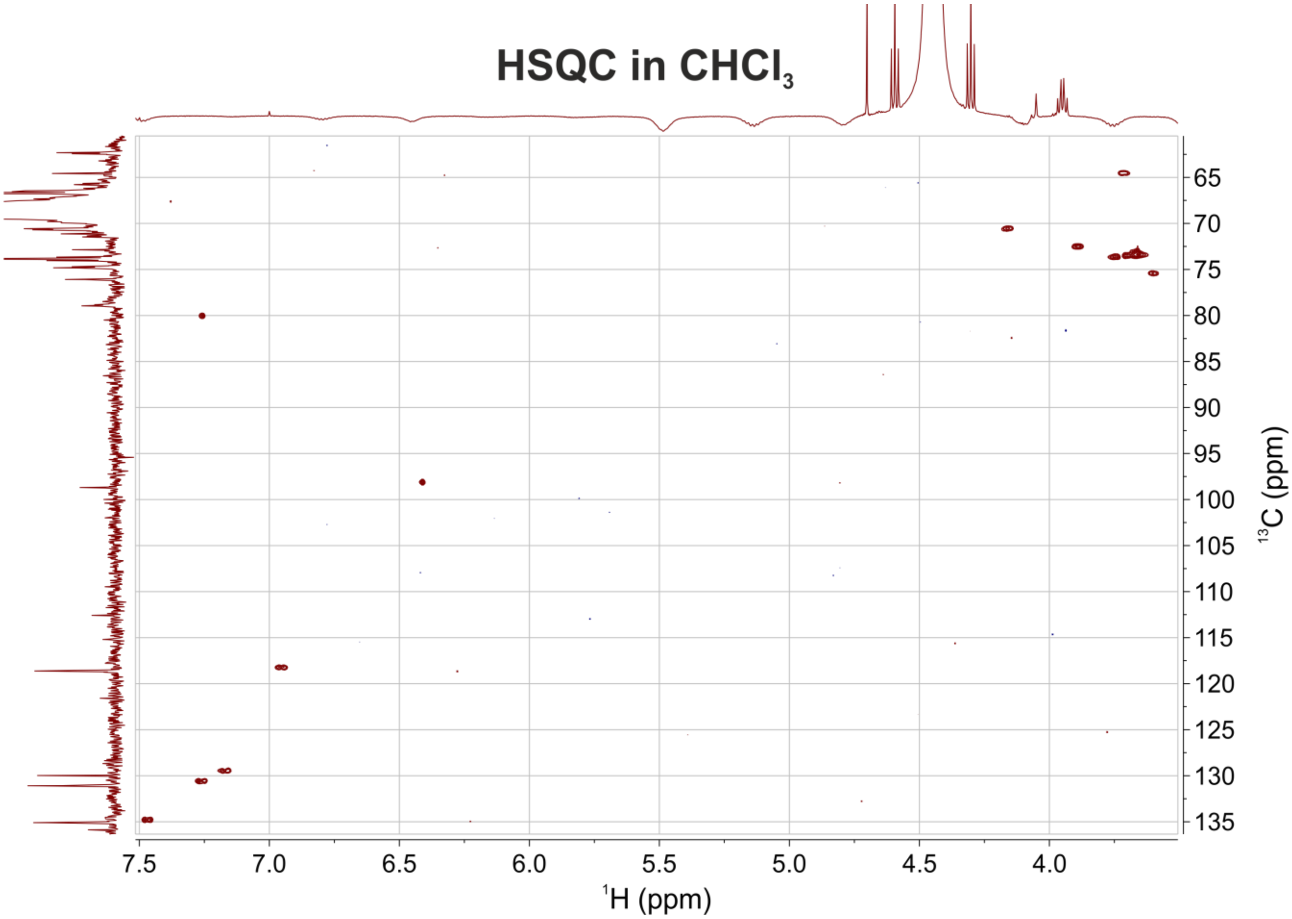

Figure S11. HSQC spectra of **2** in $CHCl_3$ with a TSP-d_4-Na salt in D_2O as a reference introduced into an NMR sample tube with a glass capillary.

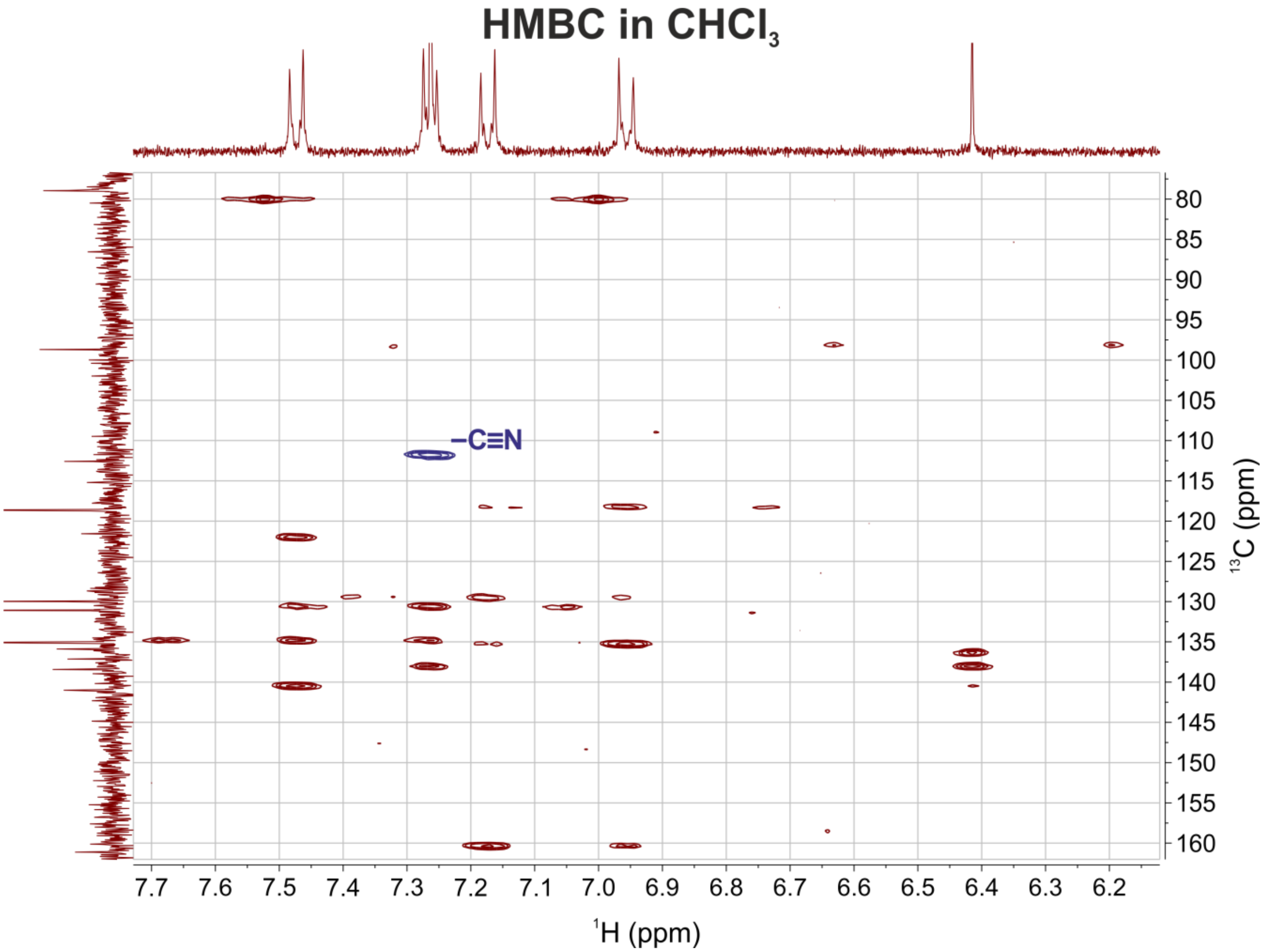

Figure S12. HMBC spectra of **2** in $CHCl_3$ with a TSP-d_4-Na salt in D_2O as a reference introduced into an NMR sample tube with a glass capillary.

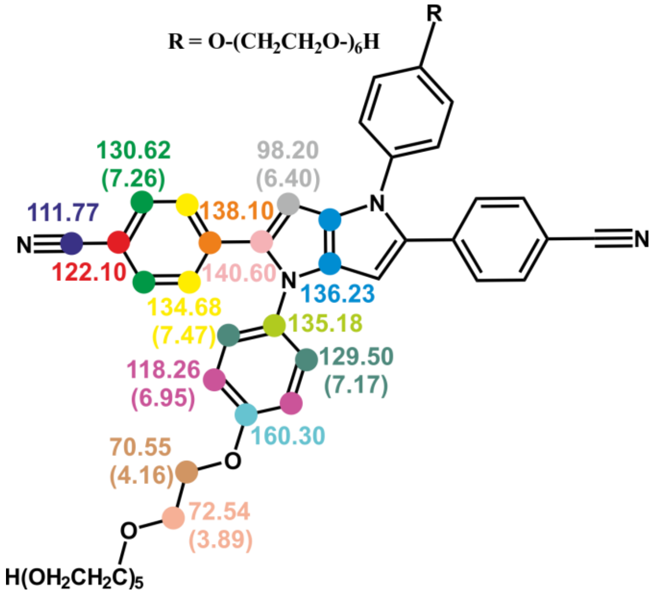

Figure S13. Assignments of ^{13}C (^{1}H) chemical shifts of **2** in $CHCl_3$ based on 2D NMR results. Only unique signals for the left half of the molecule are shown.

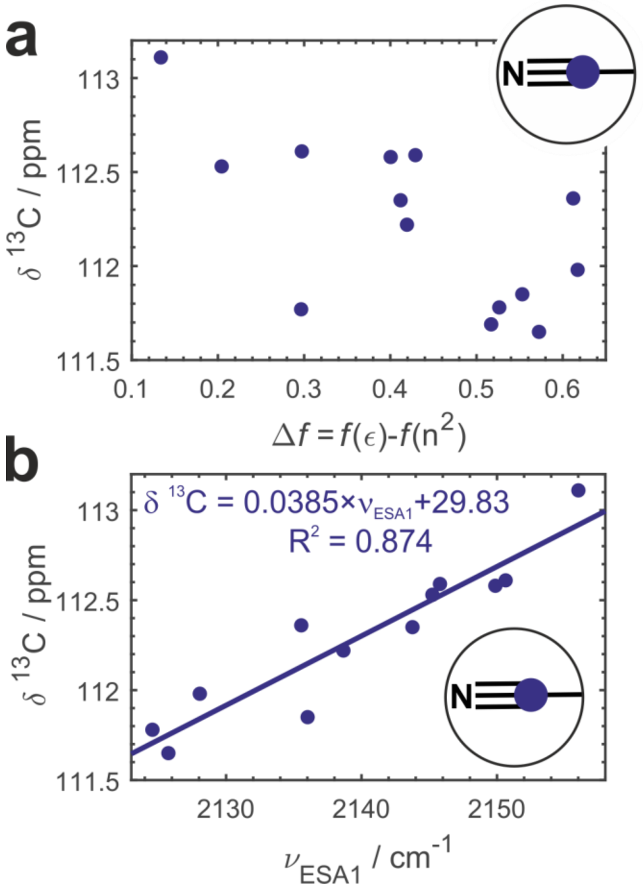

Figure S14. a. Dependence of the nitrile ^{13}C chemical shift on Onsager dipolar polarizability function. Note that the correlation is weak pointing that Onsager model does not work well in this case. In contrast, panel **b** plots the nitrile chemical shift vs. ESA1 peak position – our preferred field metric. The linear correlation is much better. Regression equation and R^2 coefficient are shown.

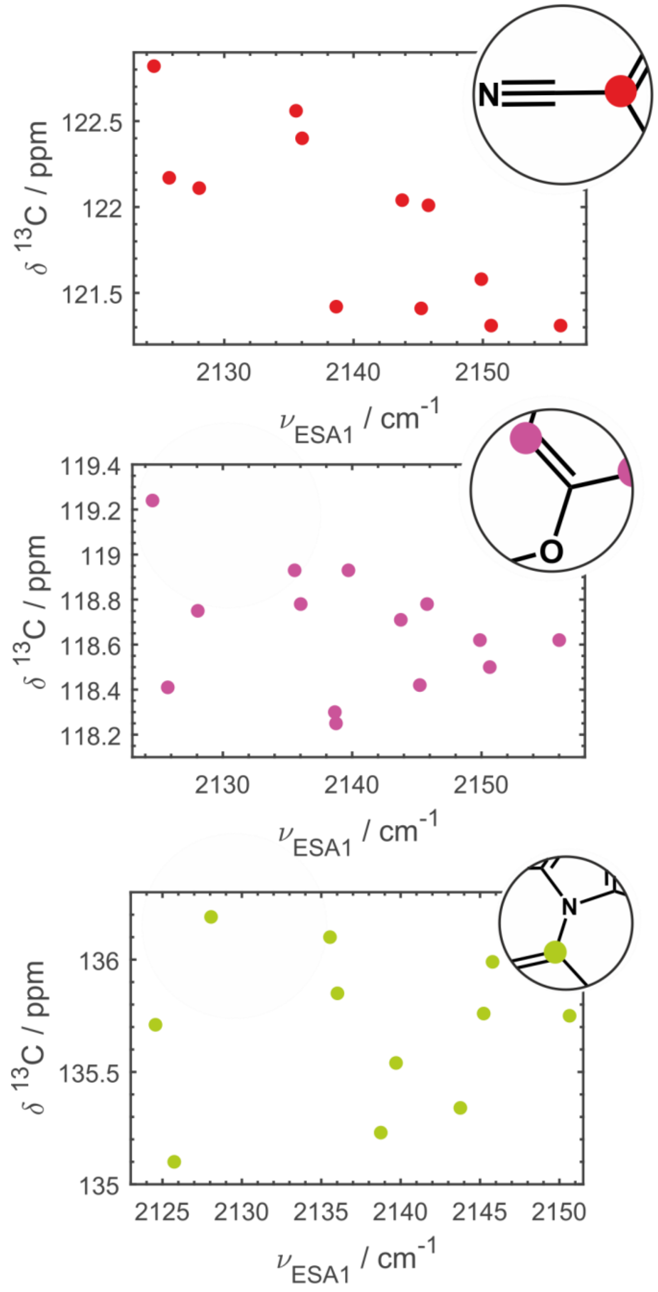

Figure S15. Dependence of several other exemplary ^{13}C resonances located in spectral vicinity of the nitrile ^{13}C carbon peak on ESA1 band position that quantifies microscopic electric field.

S3 SUPPLEMENTARY REFERENCES

(S1) Tasior, M.; Koszarna, B.; Young, D. C.; Bernard, B.; Jacquemin, D.; Gryko, D.; Gryko, D. T. Fe(III)-Catalyzed Synthesis of Pyrrolo[3,2-b]Pyrroles: Formation of New Dyes and Photophysical Studies. *Organic Chemistry Frontiers* **2019**, *6* (16), 2939–2948. https://doi.org/10.1039/C9QO00675C.

(S2) Bouzide, A.; Sauvé, G. Silver(I) Oxide Mediated Highly Selective Monotosylation of Symmetrical Diols. Application to the Synthesis of Polysubstituted Cyclic Ethers. *Org Lett* **2002**, *4* (14), 2329–2332. https://doi.org/10.1021/ol020071y.

(S3) Dereka, B.; Vauthey, E. Solute–Solvent Interactions and Excited-State Symmetry Breaking: Beyond the Dipole–Dipole and the Hydrogen-Bond Interactions. *J Phys Chem Lett* **2017**, *8* (16), 3927–3932. https://doi.org/10.1021/acs.jpclett.7b01821.

(S4) Janiga, A.; Glodkowska-Mrowka, E.; Stoklosa, T.; Gryko, D. T. Synthesis and Optical Properties of Tetraaryl-1,4-Dihydropyrrolo[3,2- b]Pyrroles. *Asian J Org Chem* **2013**, *2* (5), 411–415. https://doi.org/10.1002/ajoc.201200201.

(S5) Bredenbeck, J.; Hamm, P. Versatile Small Volume Closed-Cycle Flow Cell System for Transient Spectroscopy at High Repetition Rates. *Review of Scientific Instruments* **2003**, *74* (6), 3188–3189. https://doi.org/10.1063/1.1574605.

(S6) Hamm, P.; Kaindl, R. a; Stenger, J. Noise Suppression in Femtosecond Mid-Infrared Light Sources. *Opt Lett* **2000**, *25* (24), 1798. https://doi.org/10.1364/OL.25.001798.

(S7) Farrell, K. M.; Ostrander, J. S.; Jones, A. C.; Yakami, B. R.; Dicke, S. S.; Middleton, C. T.; Hamm, P.; Zanni, M. T. Shot-to-Shot 2D IR Spectroscopy at 100 KHz Using a Yb Laser and Custom-Designed Electronics. *Opt Express* **2020**, *28* (22), 33584. https://doi.org/10.1364/OE.409360.

(S8) Feng, Y.; Vinogradov, I.; Ge, N.-H. General Noise Suppression Scheme with Reference Detection in Heterodyne Nonlinear Spectroscopy. *Opt Express* **2017**, *25* (21), 26262. https://doi.org/10.1364/OE.25.026262.

(S9) Yanai, T.; Tew, D. P.; Handy, N. C. A New Hybrid Exchange-Correlation Functional Using the Coulomb-Attenuating Method (CAM-B3LYP). *Chem Phys Lett* **2004**, *393* (1–3), 51–57. https://doi.org/10.1016/j.cplett.2004.06.011.

(S10) Frisch, M. J.; Trucks, G. W.; Schlegel, H. B.; Scuseria, G. E.; Robb, M. A.; Cheeseman, J. R.; Scalmani, G.; Barone, V.; Mennucci, B.; Petersson, G. A.; Nakatsuji, H.; Caricato, M.; Li, X.; Hratchian, H. P.; Izmaylov, A. F.; Bloino, J.; Zheng, G.; Sonnenberg, J. L.; Hada, M.; Ehara, M.; Toyota, K.; Fukuda, R.; Hasegawa, J.; Ishida, M.; Nakajima, T.; Honda, Y.; Kitao, O.; Nakai, H.; Vreven, T.; Montgomery, Jr., J. A.; Peralta, J. E.; Ogliaro, F.; Bearpark, M.; Heyd, J. J.; Brothers, E.; Kudin, K. N.; Staroverov, V. N.; Kobayashi, R.; Normand, J.; Raghavachari, K.; Rendell, A.; Burant, J. C.; Iyengar, S. S.; Tomasi, J.; Cossi, M.; Rega, N.; Millam, J. M.; Klene, M.; Knox, J. E.; Cross, J. B.; Bakken, V.; Adamo, C.; Jaramillo, J.; Gomperts, R.; Stratmann, R. E.; Yazyev, O.; Austin, A. J.; Cammi, R.; Pomelli, C.; Ochterski, J. W.; Martin, R. L.; Morokuma, K.; Zakrzewski, V. G.; Voth, G. A.; Salvador, P.; Dannenberg, J. J.; Dapprich, S.; Daniels, A. D.; Farkas, Ö.; Foresman, J. B.; Ortiz, J. V.; Cioslowski, J.; Fox, D. J. Gaussian 09 (Revision D.1). Gaussian, Inc.: Wallingford CT 2009.

(S11) Bernetti, M.; Bussi, G. Pressure Control Using Stochastic Cell Rescaling. *J Chem Phys* **2020**, *153* (11). https://doi.org/10.1063/5.0020514.

(S12) Bussi, G.; Donadio, D.; Parrinello, M. Canonical Sampling through Velocity Rescaling. *J Chem Phys* **2007**, *126* (1). https://doi.org/10.1063/1.2408420.

(S13) Essmann, U.; Perera, L.; Berkowitz, M. L.; Darden, T.; Lee, H.; Pedersen, L. G. A Smooth Particle Mesh Ewald Method. *J Chem Phys* **1995**, *103* (19), 8577–8593. https://doi.org/10.1063/1.470117.

(S14) Hess, B.; Bekker, H.; Berendsen, H. J. C.; Fraaije, J. G. E. M. LINCS: A Linear Constraint Solver for Molecular Simulations. *J Comput Chem* **1997**, *18* (12), 1463–1472. https://doi.org/10.1002/(SICI)1096-987X(199709)18:12<1463::AID-JCC4>3.0.CO;2-H.

(S15) Van Der Spoel, D.; Lindahl, E.; Hess, B.; Groenhof, G.; Mark, A. E.; Berendsen, H. J. C. GROMACS: Fast, Flexible, and Free. *J Comput Chem* **2005**, *26* (16), 1701–1718. https://doi.org/10.1002/jcc.20291.

(S16) Stancik, A. L.; Brauns, E. B. A Simple Asymmetric Lineshape for Fitting Infrared Absorption Spectra. *Vib Spectrosc* **2008**, *47* (1), 66–69. https://doi.org/10.1016/j.vibspec.2008.02.009.
